# Liver and Muscle Transcriptomes Differ in Mid-Lactation Cows Divergent in Feed Efficiency in the Presence or Absence of Supplemental Rumen-Protected Choline

**DOI:** 10.3390/metabo13091023

**Published:** 2023-09-19

**Authors:** Malia J. Caputo, Wenli Li, Sophia J. Kendall, Anna Larsen, Kent A. Weigel, Heather M. White

**Affiliations:** 1 Department of Animal and Dairy Sciences, University of Wisconsin-Madison, Madison, WI 53706, USA; mmartin37@wisc.edu (M.J.C.); sophia.kendall@wisc.edu (S.J.K.); alarsen6@wisc.edu (A.L.); kent.weigel@wisc.edu (K.A.W.); 2 United States Department of Agriculture-Agriculture Research Station, Madison, WI 53706, USA; wenli.li@usda.gov

**Keywords:** residual feed intake, RNAseq, oxidative stress, fatty acids, metabolism

## Abstract

Improving dairy cow feed efficiency is critical to the sustainability and profitability of dairy production, yet the underlying mechanisms that contribute to individual cow variation in feed efficiency are not fully understood. The objectives of this study were to (1) identify genes and associated pathways that are altered in cows with high- or low-residual feed intake (RFI) using RNA sequencing, and (2) determine if rumen-protected choline supplementation during mid-lactation would influence performance or feed efficiency. Mid-lactation (134 ± 20 days in milk) multiparous Holstein cows were randomly assigned to either supplementation of 0 g/d supplementation (CTL; *n* = 32) or 30 g/d of a rumen-protected choline product (RPC; 13.2 g choline ion; *n* = 32; Balchem Corp., New Hampton, NY, USA). Residual feed intake was determined as dry matter intake regressed on milk energy output, days in milk, body weight change, metabolic body weight, and dietary treatment. The 12 cows with the highest RFI (low feed efficient; LE) and 12 cows with the lowest RFI (high feed efficient; HE), balanced by dietary treatment, were selected for blood, liver, and muscle analysis. No differences in production or feed efficiency were detected with RPC supplementation, although albumin was greater and arachidonic acid tended to be greater in RPC cows. Concentrations of β-hydroxybutyrate were greater in HE cows. Between HE and LE, 268 and 315 differentially expressed genes in liver and muscle tissue, respectively, were identified through RNA sequencing. Pathway analysis indicated differences in cell cycling, oxidative stress, and immunity in liver and differences in glucose and fatty acid pathways in muscle. The current work indicates that unique differences in liver and muscle post-absorptive nutrient metabolism contribute to sources of variation in feed efficiency and that differences in amino acid and fatty acid oxidation, cell cycling, and immune function should be further examined.

## 1. Introduction

Feed is the single greatest expense on a dairy farm, representing approximately half the cost of dairy production in the United States [1]. One way to thwart rising feed costs is to improve feed efficiency, which can improve the profitability and sustainability of dairy production. Proposed by Koch et al. [2], residual feed intake (RFI) is a measure of feed efficiency defined as the difference between observed and expected feed intake, such that negative RFI indicates an efficient cow that is consuming less than expected, whereas positive RFI indicates an inefficient cow that is consuming more than expected. Residual feed intake accounts for known energy sinks (i.e., milk energy output, metabolic body weight, and body weight change) for a group of cows managed similarly [3]; therefore, RFI is independent of production and body size and represents the unexplained variation in feed intake. In Holstein dairy cattle, RFI is a heritable trait (h^2^ = 0.14 to 0.25) that can be used to select animals for improved feed efficiency [4,5,6,7,8].

Although RFI is a valuable tool that enables selection for greater feed efficiency, the underlying mechanisms that contribute to RFI are not fully understood. Previous work attributed the variation in RFI to six major biological processes: feeding behavior, digestibility, metabolism (including anabolism, catabolism, and variation in body composition), heat increment of fermentation, physical activity, and thermoregulation [9]. Of these, metabolism represents nearly half of the variation in RFI in beef cattle [9]. In dairy cattle, the plasma profile of metabolites differed between more feed efficient (lower RFI) and less feed efficient (higher RFI) cows, most notably in amino acids and acylcarnitines, which indicated differences in amino acid and fatty acid metabolism between the feed efficiency groups [10]. In a respiration chamber study, early lactation cows with higher energy corrected milk (ECM)/dry matter intake (DMI) had reduced fatty acid oxidation per unit of metabolic body weight (BW) compared with cows with lower ECM/DMI [11]. While both studies indicate differences in post-absorptive nutrient metabolism, the tissue(s) and metabolic pathways responsible for the differences were not determined. Considering that liver and muscle are the primary tissues known to metabolize amino acids and fatty acids, these tissues are key targets for future research in this area.

In addition to individual cow variation in basal metabolism and nutrient use efficiency, there may also be interactions with nutrient intake. Rumen-protected choline is a nutrient that has been shown to increase DMI and ECM in early lactation when added to the diet from 3 weeks prepartum through 8 weeks postpartum [12]. In vitro work with choline chloride [13] and in vivo work with rumen-protected choline [14] have shown hepatic increases in very-low density lipoprotein, supporting triglyceride export and milk fat production. Supplementation of choline chloride in vitro has indicated increased hepatic gluconeogenic capacity by altering gene expression and increasing glycogen [15]. Increased glycogen has also been observed with rumen-protected choline supplementation in vivo [16,17]. In addition to improving nutrient metabolism, rumen-protected choline may mitigate inflammation in the postpartum period [18].

Although there is abundant evidence on the effects of supplementing choline in the transition period, there is a dearth of evidence regarding the effects of rumen-protected choline supplementation in later stages of lactation. Considering the postpartum milk production and DMI intake response, and the role choline plays in the liver, choline may influence feed efficiency in mid-lactation. Concentrations of plasma choline were lower and concentrations of plasma betaine were higher in more feed efficient (lower RFI) mid-lactation cows compared with less feed efficient (higher RFI) cows, indicating a potential differential supply or metabolism of choline between animals differing in feed efficiency status [10]. Given the lower concentrations of choline in more feed efficient animals, it is possible that efficient animals may benefit from supplemental rumen-protected choline.

Understanding the contribution of post-absorptive nutrient metabolism to variation in RFI can provide valuable insights into how to further improve the feed efficiency of dairy cattle. Furthermore, understanding potential interactions with nutrition strategies could guide future nutritional approaches. The objectives of this study were to (1) identify genes and associated pathways that are altered in feed efficient versus inefficient cows using RNA sequencing, and (2) determine if rumen-protected choline supplementation during mid-lactation would influence performance or interact with feed efficiency.

## 2. Materials and Methods

All animal protocols were approved by the University of Wisconsin-Madison College of Agricultural and Life Sciences Animal Care and Use Committee (IACUC #A006420). Multiparous Holstein dairy cows (*n* = 64) at the University of Wisconsin-Madison Emmons Blaine Dairy Cattle Research Center (Arlington, WI, USA), between 100 and 200 days in milk (134 ± 20 days in milk), were enrolled for a 47-d study, as defined previously [6,19]. Cows were housed in a sand-bedded freestall barn equipped with electronic feeding gates (RIC system, Insentec, Markenesse, The Netherlands) and ad libitum access to water.

Cows were randomly assigned to supplementation of 0 g/d (CTL; *n* = 32) or 30 g/d of a rumen-protected choline product (RPC; 13.2 g choline ion; *n* = 32; Balchem Corp.). Feeding gates were randomly assigned to treatments (TRT) and cows had ad libitum access to feed from all gates containing their assigned diet (*n* = 16/TRT). Both TRT were fed as a total mixed ration (TMR) diet formulated to meet or exceed the requirements of a 750 kg lactating cow based on the National Research Council [20], and diet composition and nutrient analysis are presented in Table 1. The RPC product was incorporated into the protein premix and thoroughly mixed into the TMR to target a supplementation rate of 30 g/d. To control for the non-choline nutrient components of the RPC product, an equivalent amount of soybean oil and soy hulls were added to the control diet (Table 1). Ruminal protection of the RPC product was 63%, as determined by a commercial lab (Cumberland Valley Analytical Services, Waynesboro, PA, USA) using an 8 h in situ incubation. The TMR was mixed once daily at 0900 h and distributed thrice daily at 1000 h, 1500 h and 2000 h. Refusals were targeted at 10% and were emptied daily, before morning feeding.

### 2.1. Sample Collection and Analysis

Samples of individual feedstuffs and TMR were collected weekly. Weekly TMR and individual feed samples were dried for 24 h in a 105 °C forced-air oven to determine dry matter (DM). Weekly samples of individual feeds were dried at 55 °C in a forced-air oven for 48 h, ground to pass a 1 mm screen (Wiley mill, Arthur H. Thomas Company, Philadelphia, PA, USA), and composited for nutrient composition analysis. Individually composited feed samples were analyzed at a commercial laboratory (Dairyland Laboratories Inc., Arcadia, WI, USA) for DM determined by National Forage Testing Association method 2.1.4 with drying for 3 h at 105 °C [22]; crude protein [23] (method 990.03); neutral detergent fiber [24] (method 2002.04); acid detergent fiber [25] (method 973.18); lignin [25] (method 973.18); ether extract [23] (method 920.39); ash [23] (method 942.05); water-soluble carbohydrates [26]; and starch was determined according to the modified procedure from Bach and Knudsen [27], in which glucose was analyzed by YSI 2700 (YSI Biochemistry Analyzer; YSI Inc., Yellow Springs, OH, USA). Total mixed ration nutrient profile was determined by averaging the nutrient profile of actual daily ingredient inclusion in the diet on a DM basis. Daily intakes were recorded electronically through electronic feeding gates.

Cows were milked twice daily at 0400 and 1500 h. Individual cow milk yield was recorded electronically at each milking, and milk samples were taken weekly at 4 consecutive evening and morning milkings. Milk samples were preserved with 2-bromo-2-nitropropane-1,3-diol (Advanced Instruments, Norwood, MA, USA), and sent to a commercial laboratory (AgSource, Menominee, WI, USA) for analysis of milk fat, true protein, lactose, and MUN by Fourier transform infrared spectrometry using the FOSS MilkoScan FT6000 (FOSS Analytical, Hilleroed, Denmark). Energy content of the milk (MilkE, Mcal/d) was determined using the equation derived from the NRC [20] Equation 2–15: MilkE = [(9.29 × milk fat (kg)) + (5.63 × true protein (kg)) + (3.95 × lactose (kg))], with the coefficient for true protein instead of crude protein. Similarly, 3.5% fat-corrected milk (FCM) was calculated as [(0.4324 × milk yield (kg)) + (16.216 × milk fat (kg))], and ECM was calculated as [(0.327 × milk yield (kg)) + (12.95 × milk fat (kg)) + (7.65 × milk protein (kg))] [28].

Body weights were recorded for three consecutive days at the beginning, middle, and end of the trial period. Body weights within a week had a coefficient of variation less than 5%. Daily BW change was calculated using the LINEST function in Microsoft Excel (Microsoft Corp., Redmond, WA, USA) of each BW and date measured. Metabolic BW (MBW) was calculated as BW^0.75^. During the same weeks as BW, body condition scores were independently recorded by the same two trained individuals using a 5-point scale with quarter-point increments [29].

#### 2.1.1. RFI Calculation

Dry matter intake for each cow was computed as a function of major energy sinks using the lm function in R 4.2.1 (R Core Team, 2022). The RFI model was:

DMI = μ + MilkE + β2 × MBW+ β3 × ΔBW + β4 × DIM + RFI,

where DMI was the observed DMI (kg), μ is the overall mean, and MilkE, MBW, ΔBW, and DIM are secreted milk energy, metabolic BW, daily change in BW, and DIM, respectively, with corresponding regression coefficients β1, β2, β3, and β4. The random residual, RFI, was considered as the feed efficiency phenotype in subsequent analyses.

The 12 cows with the highest and 12 cows with the lowest RFI, balanced by TRT, were identified as low feed efficient (LE) and high feed efficient (HE), respectively. The R^2^ of the final RFI model was 0.64 and the root mean squared error was 1.28 kg/d. Secreted milk energy and metabolic BW explained a significant (*p* < 0.001) portion of the variation in DMI, whereas DIM (*p* = 0.73) and change in BW (*p* = 0.11) did not. The model estimates (standard error) were DMI = 4.902 (3.487) + 0.332 (0.048) × MilkE + 0.107 (0.019) × MBW + 0.705 (0.439) × ΔBW + −0.003 (0.009) × DIM.

#### 2.1.2. Blood Sample Collection and Metabolite Quantification

Blood samples were collected from the coccygeal vessel from all cows in the final week of the trial period after the morning milking and before morning feeding. Blood samples were taken across four days, with cows being assigned randomly to sampling day and balanced by TRT and midpoint RFI (Supplementary Materials). Blood samples were collected in tubes containing potassium oxalate and 4% sodium fluoride (BD Vacutainer; BD, Franklin Lakes, NJ, USA) and tubes containing ethylenediaminetetraacetic acid dipotassium salt dihydrate for plasma separation (BD Vacutainer; BD, Franklin Lakes, NJ, USA), and tubes without preservative for serum separation. Plasma was separated from whole blood by centrifugation at 2000× *g* at 4 °C for 15 min, then immediately aliquoted into tubes and stored at −20 °C or −80 °C until analysis. Serum was separated from whole blood by centrifugation at 2500× *g* at 20 °C for 15 min, then immediately aliquoted into tubes and stored at −20 °C until analysis.

Quantification of plasma β-hydroxybutyrate (BHB), glucose, blood urea nitrogen (BUN), creatinine, alanine aminotransferase (ALT) and aspartate aminotransferase (AST) were based on previously described chemistries and Catachem VETSPEC reagents and determined using the Catachem Well-T AutoAnalyzer (Catachem, Oxford, CT, USA) as detailed in the Supplemental Materials. Plasma concentrations of triglyceride, fatty acids, and bilirubin were quantified using Catachem VETSPEC reagents in modified protocols and fatty acids and bilirubin were quantified using a Synergy H1 Hybrid Spectrophotometer (BioTek, Winooski, VT, USA), as detailed in the Supplementary Materials. Serum insulin concentrations were quantified using bovine ELISA kits (Mercodia Immunoassays and Services, 10–1201–01, Uppsala, Sweden) according to the manufacturer’s protocol. Briefly, insulin reacted with peroxidase-conjugated anti-insulin antibodies and anti-insulin antibodies that were bound to the well. The sample was washed, and the bound conjugate reacted with 3,3′5-5′tetramethylbenzidine. The reaction was halted with the addition of acid and the reaction was read at 450 nm using a Synergy H1 Hybrid Spectrophotometer (BioTek, Winooski, VT, USA). Reference pool samples of each sample type were used for assay quality control. Inter-assay coefficients of variation were 4.4, 4.3, 7.7, 4.3, 7.7, 4.3, 3.5, 4.0, 6.3, 5.4, and 7.4% for albumin, BHB, bilirubin, BUN, creatinine, glucose, AST, ALT, fatty acids, triglyceride, and insulin, respectively. Intraassay coefficients of variation did not exceed 10%. The RQUICKI was calculated as: RQUICKI = (log [glucose mg/dL] + log [insulin µIU/mL] + log [NEFA mmol/L]) after converting insulin to international units [30,31].

Blood fatty acids were extracted and methylated from plasma using a modified method from Sukhija and Palmquist [32]. Briefly, plasma was added to 5% methanolic hydrochloric acid and chloroform spiked with C19:0 (74208; Sigma-Aldrich, St. Louis, MO, USA), placed in a 70 °C water bath for 2 h, then neutralized with potassium carbonate. The chloroform layer was removed and evaporated under nitrogen until approximately 200 μL remained. If the chloroform layer was <200 μL, chloroform was added to a final volume of approximately 200 μL. Plasma fatty acids were determined utilizing gas-liquid chromatography with a mass spectrometer (GC690 and Clarus SQ 8T; PerkinElmer, Waltham, MA, USA) using a 100 m capillary column (J&W CP-Sil 88 fused silica, 100 m × 0.25 mm column, Agilent, Santa Clara, CA, USA). The injection volume was 1 μL with a 10:1 split. Helium was used as a carrier gas with a flow of 2.0 mL/min. The injector temperature was 230 °C, the GC inlet temp was 200 °C, and the MS source was 230 °C. The oven temperature was 50 °C for 1 min, ramped up to 175 °C at a rate of 15 °C/min, then ramped up to 210 °C at 1 °C/min and held at 210 °C for 5 min. The MS used electron ionization with a scan mode ranging from 50 to 430 *m/z* and a solvent delay of 11 min. Peaks were identified based on commercially available individual FAME standard mixtures: FIM- FAME-5 and -6, (4210 and 2009; Matreya Inc., State College, PA, USA). Peaks were analyzed in TurboMass (v. 6.1.2.2024; PerkinElmer, Waltham, MA, USA) using C:19 as an internal standard.

#### 2.1.3. Liver and Muscle Tissue Collection and Analysis

Biopsies were collected across 4 days, with tissue samples paired to the same day as the blood sample. Biopsies were collected between 2 and 6 h after morning feeding. Liver tissue samples (~1 g) were obtained by blind percutaneous biopsy utilizing custom trocars [33,34,35,36]. Semitendinosus muscle tissue samples (~1 g) were obtained using a biopsy punch as detailed in the Supplementary Materials. Immediately after collection, tissue samples were rinsed with saline, aliquoted into RNA-free tubes containing 1 mL of TRIzol (15596018; Thermo Fisher Scientific, Carlsbad, CA, USA) flash frozen in liquid nitrogen, and stored at −80 °C until analysis.

Liver and muscle tissue RNA were isolated as described previously [36]. Briefly, total RNA was extracted from ~200 g of tissue using a phenol, chloroform extraction [37] and then purified utilizing the Aurum Total RNA 96 Kit (7326800; Bio-Rad Laboratories, Hercules, CA, USA) with previously described modifications [36]. Total RNA was quantified using a Synergy H1 Hybrid Spectrophotometer (BioTek, Winooski, VT, USA) as described [36]. Absorbance of 260/280 nm of total RNA was between 2.11 and 2.15 for liver and between 1.85 and 2.16 for muscle. Due to low RNA concentrations, only 16 muscle tissue samples (*n* = 8/ RFI group; *n* = 7 CTL, *n* = 9 RPC) were utilized for further analysis. Liver and muscle RNA was further assessed using the Bioanalyzer 2100 (Agilent Technologies, Santa Clara, CA, USA) to obtain RNA integrity numbers (mean ± SD; Liver = 7.2 ± 0.8; Muscle = 7.4 ± 0.6).

Library preparation for RNA sequencing was performed using the Illumina Stranded Total RNA Prep with Ribo-Zero Plus (20040525; Illumina, San Diego, CA, USA) with IDT for Illumina RNA UD Indexes Set A (20040553; Illumina, San Diego, CA, USA). A Sciclone G3 (PerkinElmer, Waltham, MA, USA) liquid handler was used for the library preparation procedure following the certified protocol by the manufacturer. For each sample, 1.0 μg of liver total RNA and 0.7 μg of muscle total RNA were used for library preparation. The fragment distribution and quantity of prepared libraries was assessed via 4200 Tape Station System (Agilent, Santa Clara, CA, USA). Pooled libraries were initially sequenced using MiSeq Nano kit (Cat. MS-102-2002; Illumina, San Diego, CA, USA). Pooling was further normalized using the index ratio generated for each sample from the MiSeq run to ensure equal quantities before sequencing. Finally, pooled libraries were sequenced on a NovaSeq 6000 instrument (Illumina, San Diego, CA, USA) to obtain paired-end, 2 × 150 bp reads. Quality of reads was assessed via FastQC (https://www.bioninformatics.babraham.ac.uk/projects/fastqu/ (accessed on 4 April 2023)). Before sequence alignment, raw reads were filtered to remove those shorter than 50 bp. Sequence alignment was performed using STAR (2.5.2b) [38]. The reference files *Bos taurus* (release 106, ARS-UCD 1.2) were downloaded from NCBI (https:/www.ncbi.nlm.nih.gov/assembly/GCF_002263795.1 (accessed on 4 April 2023)). Differential gene expression analysis was conducted in DESeq2 [39] using gene-level raw read counts generated by STAR as the input. To determine significantly differentially genes, combinatory cutoff values were used as follows: gene mean read count ≥10, fold-change ≥1.5 and *p*-value < 0.01. Expression level of mRNAs in each sample were calculated as the normalized mean read count (NMRC) using the median of ratios method [40] using DESeq2.

### 2.2. Statistics

One cow on RPC was removed from the analysis due to health reasons unrelated to dietary treatment, and two cows on CTL were removed due to consuming >10% of average daily DMI from the RPC treatment, as determined by the reports from the electronic feeding gates. Final analysis included *n* = 30 CTL and *n* = 31 RPC. Data analysis was performed using the PROC GLIMMIX and PROC MIXED procedures of SAS (version 9.4, SAS Institute Inc., Cary, NC, USA). The independent effects of TRT (CTL or RPC) or RFI group (LE or HE) on production responses were determined using linear models with fixed effect of the TRT or RFI group, respectively (*n* = 61). The effects of the RFI group and TRT on blood metabolites were determined using linear models with fixed effects of the RFI group, TRT, and their interaction, with a random effect of day of sampling (*n* = 24). Studentized residuals were assessed visually by plotting (linear predictor × studentized residuals, studentized residual quantile-quantile plot, and effect × studentized residuals plots) to determine if studentized residuals had equal variance, zero mean, and normal distribution. If these model assumptions were not met, a Box-Cox transformation was used. When model assumptions could not be met through traditional normalization methods and unequal variance persisted, models with heterogeneous variance were used, and the model with the lowest Akaike’s information criteria that improved studentized residual plots was selected. Fixed effects with a *p* ≤ 0.05 were considered to have significant evidence for differences, and effects with a 0.05 < *p* ≤ 0.10 were considered to have a tendency for significant differences. When a RFI group × TRT effect had tendency for significance (*p* ≤ 0.10), pairwise comparisons were made with corrections for multiplicity using Tukey’s honest significant difference. Data are presented as the least square means and 95% confidence intervals (mean [95% confidence interval).

Differential gene expression analysis was performed in DESeq2 [39] in R. Differentially expressed genes (DEG) were genes with *p* ≤ 0.05, fold change ≥1.5, and NMRC ≥ 10. Highly expressed DEG were DEG with NMRC > 200. The Database for Annotation, Visualization, and Integrated Discovery (DAVID; v.2021) software was used to evaluate gene ontologies (GO) and Kyoto Encyclopedia of Genes and Genomes (KEGG) pathways for DEG. The gene list was compared to the *Bos taurus* genome. Differences were corrected using Benjamini–Hochberg false discovery rate adjusted *p*-value [41]. Pathways and GO terms with *p* ≤ 0.05 were considered to have significant evidence for differences, and effects with a 0.05 < *p* ≤ 0.10 were considered to have a tendency for significant differences.

## 3. Results

### 3.1. High and Low RFI Grouping

Least squares means for performance variables by RFI group are reported in Table 2. As expected, milk energy output, metabolic BW, and BW change were not different between low feed efficient (LE) and high feed efficient (HE) cows (*p* ≥ 0.50). Dry matter intake was 4 kg lower in HE cows (29.5 kg [28.5, 30.6] vs. 33.5 kg [32.5, 34.6] kg) with a 3.5 kg range in RFI (HE: −1.73 kg; LE: 1.72 kg).

### 3.2. Production Response of RPC Supplementation

On average, cows supplemented with RPC consumed (mean ± SD) 28.3 ± 2.2 g/d of the rumen-protected choline product (13.0 ± 1.0 g/d choline ion) and CTL cows consumed 0.0 ± 0.008 g/d of the rumen-protected choline product (0.0 ± 0.004 g/d choline ion). The least squares means and confidence intervals of performance variables by TRT are presented in Table 3. Supplementing RPC did not alter milk production, ECM, DMI, or BW (*p* > 0.25). Supplementing RPC tended (*p* = 0.07) to increase MUN (14.7 mg/dL [14.2, 15.2] vs. 14.1 mg/dL [13.6, 14.5]), but not other milk components (*p* > 0.10). Feed efficiency was unaffected by treatment (*p* = 0.76).

### 3.3. Blood Metabolites

The blood metabolite results for the top 12 (LE) and bottom 12 (HE) cows of RFI are presented in Table 4 and Table 5. Effects of RPC on blood metabolites, based on results from all cows (*n* = 60), are presented in Supplementary Tables S1 and S2. There was no evidence for an effect of TRT or RFI group on fatty acids, bilirubin, BUN, creatinine, AST, insulin, or RQUICKI (*p* > 0.10; Table 4). Plasma BHB concentrations were greater in HE cows compared with LE cows (0.71 mmol/L [0.60, 0.85] vs. 0.59 mmol/L [0.49, 0.70]; *p* = 0.04; Table 4). Cows receiving RPC treatment had greater albumin concentrations (3.84 g/dL [3.75, 3.95] vs. 3.70 g/dL [3.62, 3.78]; *p* = 0.03) compared with CTL. There was a tendency (*p* = 0.09) for an interaction with glucose concentrations; LE cows supplemented with RPC had greater glucose concentrations than LE cows receiving CTL (Table 4). There was an interaction (*p* = 0.05) with triglyceride concentrations, where HE cows supplemented with RPC had lower concentrations than HE cows receiving CTL. Treatment interacted (*p* = 0.01) with RFI group for ALT activity, such that HE cows receiving RPC had lower activity than LE cows on either treatment, and tended (*p* = 0.06) to have lower activity of ALT than HE cows receiving CTL.

Eleven medium- and long-chain fatty acids were identified in plasma samples, ranging from C14:0 to C20:4 (Table 5). There was no evidence for an effect of TRT or RFI group on C15:0, C16:0, C16:1 (*cis*-9), C18:0, C18:1 (*cis*-9), C18:2 (*cis*-9,12), C18:3 (*cis*-9,12,15), and C20:3 (*cis*-11,14,17). Arachidonic acid (C20:4, *cis*-5,8,11,14) tended to be greater in RPC compared with CTL (0.76 mg/dL [0.74, 0.79] vs. 0.73 mg/dL [0.70, 0.76]; *p* = 0.09). There was an interaction in the RFI group and TRT for heptadecanoate (C17:0), with HE cows receiving RPC tending to have lower concentrations compared with HE cows receiving CTL (*p* = 0.08), and compared with LE cows receiving RPC (*p* = 0.06). There was a tendency for an interaction in the RFI group and TRT for myristate (C14:0), but individual treatment means were not different.

### 3.4. Liver and Muscle Tissue Transcriptome

**Figure 1:**
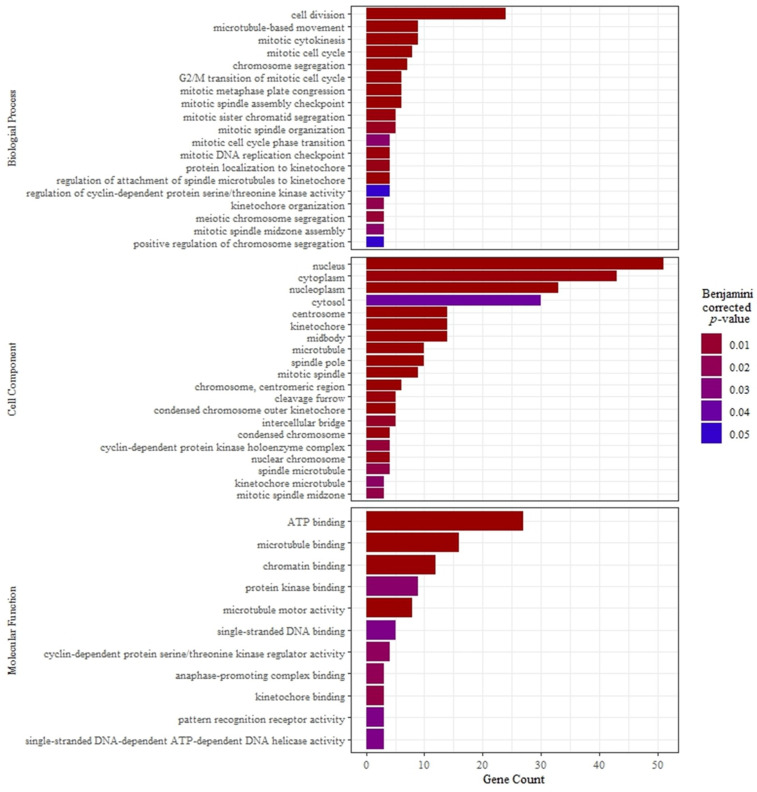
Enrichment analysis of the Gene Ontology domains was performed using the Database for Annotation, Visualization, and Integrated Discovery (v. 2021) comparing a list of differentially expressed genes (mean read count ≥ 10; *p*-value ≤ 0.05; fold change ≥ 1.5) between liver tissue of high feed efficient (HE; *n* = 12) and low feed efficient (LE; *n* = 12).

A total of 40 samples (24 liver and 16 muscle tissue), balanced by RFI group, were used for RNA sequencing. For liver samples, the mean number of total paired-end reads per library was (mean ± standard error) 70.7 ± 1.9 M, and mean mapping rate to the *Bos taurus* genome was 94.1 ± 0.1%. For muscle samples, the mean number of total paired-end reads per library was 73.9 ± 2.3 M, and mean mapping rate to the *Bos taurus* genome was 93.2 ± 0.6%. In liver tissue, 26,552 genes had NMRC > 0. Between the HE and LE groups, 268 DEG were identified in the liver, 180 DEG were upregulated and 88 DEG were downregulated in HE cows compared with LE cows (Supplementary Table S3). Four KEGG metabolic pathways were enriched in the upregulated DEG of HE cows, including the cell cycle, cell senescence, p53 signaling pathways, and Human T-cell leukemia virus 1 infection (Table 6). Of the GO domains, 19 biological processes, 20 cellular components, and 11 molecular functions differed within upregulated DEG of HE cows (Figure 1; Supplementary Table S4). Four of the 88 downregulated DEG in HE cows were involved in the biological process GO:0071280, the cellular response to copper ion (Fold enrichment = 85.5; Corrected *p*-value = 0.0001; *AQP2*, *MT1E*, *MT1A*, *SNCA*).

In muscle tissue, 24,933 genes had NMRC > 0. Between the HE and LE groups, 315 DEG were identified, of which 152 were upregulated and 163 were downregulated in HE (Supplementary Table S5). Upregulated DEG in HE cows were involved in cell component GO term, extracellular space, and molecular function GO term, actin filament binding (Table 7; Supplementary Table S6). Downregulated DEG in HE cows were involved with the cell component GO term “nucleus”.

There were 10 common DEG between liver and muscle tissue, of which five were downregulated in HE (*FANCI*, *LOC534578*, *LOC787122*, *MKI67*, and *RNASE10*), two were upregulated in HE (*LOC101905179* and *LOC101903301*), two were upregulated in muscle but downregulated in liver of HE (*BHLHE23* and *CYR61*), and *LOC100196897* was upregulated in muscle and downregulated in liver of HE compared with LE cows.

## 4. Discussion

A national concerted effort to develop genomic evaluations for dairy cattle feed efficiency has been underway to improve the feed efficiency of the dairy cattle breed in effort to reduce feed costs, increase farm profitability, and improve sustainability [42,43,44]. In tandem with genetic selection of feed efficiency, it is necessary to understand the sources of variation in RFI to inform genetic selection and anticipate correlated responses in animal health, physiology, or metabolism. Such knowledge will also aid in identifying areas of management and nutrition that can be manipulated to further improve efficiency, and it will inform the care and nutritional management of cows that are genetically predisposed to highly efficient utilization of feedstuffs. Previous work has identified post-absorptive nutrient metabolism as a source of variation in RFI [9,10,11], although further investigation is necessary to determine the tissues involved and the pathways that are altered. Within the current work, we aimed to identify liver and muscle tissue-specific genes and associated pathways that differed between HE and LE cows and determine the relationship between mid-lactation feed efficiency and RPC supplementation to deepen our understanding of post-absorptive nutrient metabolism as a source of variation in RFI.

### 4.1. Response of RPC Supplementation

Although most non-ruminant mammals have a dietary choline requirement [45,46], there is no defined choline requirement for dairy cows. Blood choline metabolites are lowest in early lactation and highest in late lactation, suggesting a need for supplemental rumen-protected choline in early lactation [47]. Additionally, the onset of negative nutrient balance during this period results in limited energy, vitamins, minerals, amino acids, and methyl donors [48,49]. Choline is commonly supplemented in the periparturient period [45] and a recent meta-analysis indicated a linear production response with RPC supplementation up to 25.2 g choline ion/d in periparturient cows [12]. The role of choline in lipid and glucose metabolism, and as a methyl donor [15,48,50], support that it may be beneficial to supplement RPC during other lactation periods; however, supplementing RPC during mid-lactation in this study did not improve production performance or feed efficiency. Previous studies supplementing RPC to mid-lactation cows in similar or greater rates than the current study observed production responses when dietary protein was low and methionine was likely deficient [51,52,53]. When the dietary crude protein was high [54] and metabolizable methionine was adequate [55], or when RPC supplementation was similar to the current study [53], no production responses were observed. In the current study, metabolizable protein was 3750 g/d and metabolizable methionine was 87.5 g/d: more than adequate for the cows’ production level and body size according to NASEM [21]. Interestingly, when mid-lactation cows were challenged by electric heat-blanket-induced heat stress, RPC supplementation before and during the challenge tended to ameliorate the effects of heat stress and milk production losses, which suggests RPC may influence nutrient use efficiency during states of metabolic challenge [56]. Response to supplementation of RPC during mid-lactation may reflect overall nutrient balance and the presence or absence of additional stressors.

Despite the lack of production responses, differences in blood metabolites were observed. Albumin, a negative acute phase protein manufactured in the liver, was elevated in cows supplemented with RPC. Hepatic inflammation can result in decreased albumin production [57], so increases in albumin concentration with RPC supplementation may indicate improved liver function and reduced inflammation, although it is only one marker of this response. Early lactation supplementation of RPC increased concentrations of blood albumin in some studies [58,59], but not others [51]. Gene expression of markers of inflammation and immune function in the liver of postpartum cows supplemented with RPC were suggestive of improved inflammatory state, which supports the reductions in plasma albumin observed here in mid-lactation cows [18].

Within the 12 most and 12 least efficient cows, arachidonic acid (C20:4, *cis*-5,8,11,14) tended to be greater in RPC supplemented cows compared with CTL. However, when including all cows (*n* = 30/TRT), arachidonic acid was not different (*p* = 0.76; Supplementary Table S2), indicating the difference by TRT was specific to cows that were most and least efficient. Arachidonic acid can come from the diet or be synthesized from linoleic acid by most mammalian cells [60]. Differences in dietary supply of arachidonic acid between TRT is unlikely given the similar intakes of an identical diet between RPC and CTL. Increased arachidonic acid could be from reduced ruminal biohydrogenation, increased synthesis from linoleic acid, or reduced utilization by the peripheral tissues. Given that the CTL diet accounted for the non-choline nutrients of the RPC product, supplementation with RPC was not likely to differentially effect rumen biohydrogenation, although rate of dissociation of the encapsulation vs. soybean oil could have introduced some variation. Arachidonic acid represents a small portion of total circulating fatty acids (2 to 3%), yet given its essential role in maintaining the structural integrity of cell membranes and prostaglandin synthesis [60,61], and potential interaction with feed efficiency, the 4% difference between RPC and CTL in arachidonic acid warrants further investigation.

### 4.2. Blood Metabolite and Tissue Difference by RFI

Hepatic oxidation of fatty acids can undergo incomplete oxidation via ketogenesis, generating ketone bodies, including BHB, which can be utilized by extrahepatic tissues for energy [62]. The generation of BHB can also originate from rumen epithelial conversion of butyrate to BHB [63]. Concentrations of BHB were elevated in HE cows compared with LE cows. Previous work in mid-lactation did not detect significant differences in BHB by RFI group, although the numeric pattern is consistent [10]. Hyperketonemia, defined as a blood BHB ≥ 1.2 mmol/L, is associated with negative outcomes during the postpartum period [64]. Average BHB concentrations were lower than this threshold in both RFI groups with no individual sample ≥1.2 mmol/L. When RFI was determined in mid-lactation and postpartum, BHB concentrations were investigated retrospectively; lower RFI cows had greater BHB concentrations [65], without differences in the incidence of hyperketonemia or other metabolic diseases [66,67,68]. Production of BHB is not inherently detrimental; instead, it represents a pathway for extrahepatic tissues to utilize alternative energetic intermediates [62]. Concentration of BHB reflects the balance of both production and utilization, and the increase observed in HE cows herein could reflect greater hepatic or ruminal production of BHB, altered utilization by peripheral tissues, or both. Future work should investigate if this difference in BHB is an adaptation in the tissue utilization of nutrients that contribute to feed efficiency status.

### 4.3. Interaction of RFI Group and TRT on Blood Metabolites

Within LE cows, increased concentrations of glucose were observed in cows supplemented with RPC. Supplementation of choline chloride in vitro suggested greater gluconeogenic capacity in hepatocytes [15], and increases in liver glycogen have been observed in vitro and in vivo when supplementing choline chloride and RPC, respectively [15,16,17]. This is further supported by observed increases in blood glucose concentrations in postpartum cows supplemented with RPC [12,14,69,70]. Circulating glucose concentrations are reflective of hepatic gluconeogenesis, extrahepatic tissue demand and utilization, and a small amount from intestinal absorption of glucose. Of the tissues that utilize glucose, the mammary gland can take up over 50% of glucose supply [71]. Lack of differences in milk yield, fat, and lactose between TRT suggests that there were no differences in mammary sequestration of glucose for lactose or glycerol synthesis, although it is possible that other tissues differed in uptake and utilization of glucose. The higher intakes in LE cows, compared with HE cows, may have resulted in an increase in gluconeogenic precursors available for hepatic gluconeogenesis, namely propionate [72]. Increased gluconeogenic precursors in LE cows, coupled with potential increased hepatic gluconeogenic capacity with RPC supplementation, may explain the increase in blood glucose concentrations observed in LE cows supplemented with RPC compared with CTL, although differences in tissue utilization cannot be disregarded.

Reduced plasma triglycerides in HE cows supplemented with RPC, compared with HE cows on CTL, indicate a specific response to RPC within more feed efficient animals. Previous work indicated HE cows may have more complete oxidation of fatty acids, without differences in plasma triglyceride, compared with LE cows [10]. Given that cows were in a state of positive energy balance and not mobilizing excessive body tissue, as evidenced by positive BW changes and low plasma fatty acid concentrations, triglycerides would be sourced primarily from the diet. Numerically, lower DMI of HE cows supplemented with RPC vs. CTL (28.8 vs. 30.3 kg) may partially explain lower concentrations of triglyceride in the RPC treatment compared with CTL in HE cows. Reduced triglycerides in HE cows supplemented with RPC vs. CTL could also originate from increased lipolysis of circulating triglycerides and increased utilization of the resultant fatty acids by tissues for milk fat synthesis, storage, or oxidation for energy. Similar yields of milk fat indicate that sequestration of fatty acids for milk fat synthesis was similar. Complete oxidation of fatty acids provides energy for the cell, and incomplete oxidation of acetyl-CoA from fatty acids in the liver can go through ketogenesis to produce BHB [62]. An increase in incomplete oxidation of fatty acids from triglyceride would support the increased BHB observed in HE cows and the numeric increase in HE cows supplemented with RPC compared with CTL.

An interaction between the RFI group and TRT was detected for ALT, in which HE cows on RPC had lower activity of ALT compared with LE cows on either TRT and tended to have lower activity of ALT compared with HE cows on CTL. Previous work in mid-lactation cows divergent in RFI without dietary interventions did not detect differences in ALT [10] or found marginal evidence for lower ALT in HE cows [73]. Activity of ALT is an indicator of hepatic damage and stress, and is associated with ketosis [74,75]. Reduced activity in HE cows supplemented with RPC may indicate improved liver function in more efficient cows, but only when supplemented with RPC. Despite this, improved liver function in HE cows is not supported by differences in other liver function markers (AST, bilirubin, and albumin) in the current study.

HE cows supplemented with RPC tended to have lower concentrations of heptadecanoic acid (C17:0) compared with HE cows on CTL and LE cows on RPC. Similarly, previous metabolomic work identified lower concentrations of heptadecanoic acid in Holstein cows [76]. Heptadecanoic acid is synthesized by ruminal bacteria with very little coming from the diet [77]. Post-ruminally, C17:0 can be synthesized in mammary and adipose tissue from propionate [77,78], but the contribution to plasma C17:0 is likely small. Tendencies for HE cows on RPC to have reduced plasma C17:0 compared with HE cows on CTL and LE cows on RPC suggest altered microbial synthesis, reduced post-absorptive synthesis, or increased post-absorptive utilization by the tissues. Although the plasma profile of odd-chain fatty acids is not perfectly representative of milk odd-chain fatty acid profile [79], C17:0 in milk decreased linearly as cows increased RPC supplementation from 21 to 63 DIM [16]. While the effect of RPC on C17:0 is not fully understood, the interaction between the RFI group and TRT warrants further investigation.

### 4.4. Liver and Muscle Transcriptome by RFI Group

Previous work comparing circulating metabolites between HE and LE mid-lactation cows found differences in amino acids, fatty acids, and acylcarnitines, suggesting that there may be differences in the pathways related to the complete oxidation of fatty acids and amino acids [10]. Analysis of the liver and muscle transcriptome in the most and least feed efficient cows herein provides novel insights into tissue-level metabolic differences that can contribute to whole-animal feed efficiency. Of the DEG in liver and muscle, pathways related to oxidative stress, immune response, and the metabolism of lipids and glucose are of particular interest for discussion here.

#### 4.4.1. Liver Transcriptome

Oxidative stress is inevitable in aerobic organisms, originating from reactive oxygen species (ROS) from mitochondrial oxidative phosphorylation, ionizing radiation exposure, metabolism of exogenous compounds, and disruptions of metabolic processes in disease [80]. Without intervention, oxidative stress can lead to DNA and cell damage or death [62]. Cells have evolved mechanisms to protect against the damaging effects of oxidative stress, including antioxidants, oxidant defense enzymes, and cell cycle checkpoint systems [80]. Progression through the cell cycle is controlled through a series of checkpoints, which prevents damaged cells from proliferating. Presence of ROS can inhibit progression through cell cycle checkpoints, allowing for damage repair or apoptosis signaling [80]. Upregulation of DEG involved in the cell cycle (KEGG: bta04110) and several related GO biological processes in the liver of HE cows compared with LE cows is suggestive of less interrupted cell cycle progression, increased cell proliferation, and reduced oxidative stress. Further evidence of reduced oxidative stress is indicated by cellular senescence (KEGG: bta04218) and p53 signaling pathways (KEGG: bta04115) enriched within upregulated DEG in the liver of HE cows compared with LE cows. The p53 pathway is activated by stress signals that impact cell homeostasis and cycling, and initiates cell cycle arrest, cellular senescence, or apoptosis [81]. Upregulation of the genes involved in the p53 signaling pathway and cellular senescence suggests a downregulation of p53 pathway activation and reduced cellular senescence in HE cows. Finally, highly expressed genes in the liver related to anti-oxidation (*MT1A*, *GPX3*, and *GSTP1*) were downregulated in HE cows compared with LE cows. These pathway shifts are in agreement with reduced plasma ALT activities in the subset of HE cows supplemented with RPC and support reduced oxidative stress and improved liver function. Other liver function markers, i.e., AST, albumin, and bilirubin, were not different between RFI groups, which could indicate that they are less sensitive oxidative markers, or that they are generally reflective of other non-oxidative stimulated liver damage. Regardless, this is an intriguing potential difference between cows with divergent feed efficiency.

The association of HE status with reduced oxidative stress was observed previously in other species, including skeletal muscle mitochondria of pigs [82], duodenal mitochondria of broilers [83], liver of broilers [84] and beef steers [85]. Oxidative stress-induced molecule or cell damage is energetically expensive to either repair or degrade and replace [86]. Reduced energetic cost from reduced oxidative stress may represent one mechanism that contributes to improved efficiency. Mitochondria are a major site of oxidation and are responsible for around 90% of ROS generation [87]. In addition to the energetic cost associated with oxidative stress, damage caused by ROS can lead to compromised mitochondrial function [88], representing an additional loss of efficiency. Previous work in broilers [83,89] and beef cattle [90,91] has shown that HE animals have improved mitochondrial function. Taken together, this collective work suggests that a source of variation in feed efficiency is oxidative stress, with the energetic cost of repairing oxidative damage and reduced mitochondria function as the consequences. Mitochondrial function analysis was not performed in the current study, but is warranted in future work to determine if it contributes to variation in feed efficiency of lactating dairy cows.

Several highly expressed genes involved in the activation of the innate and adaptive immune system response were upregulated in HE cows (*LYZ*, *AOAH*, *CLEC7A*, *GIMAP8*, *GIMAP6*, *MARCO*, and *CD180*) and one was downregulated in HE cows (*SAA4*). The *AOAH* gene encodes acyloxyacyl hydrolase (AOAH), a lipase that removes secondary fatty acids of lipopolysaccharide (LPS), inactivating LPS [92]. Gene expression of *AOAH* is increased when mice are exposed to LPS [93], and AOAH can prevent harmful consequences of the inflammatory response [92]. Gene expression of *MARCO* and *LYZ*, markers of pro-inflammatory macrophage activation, are also increased after LPS exposure [94,95] and increased expression of *MARCO* can be interpreted as a sign of macrophage activation [94]. The protein encoded by *CLEC7A* recognizes the cell wall 1,3-β-glucans of fungi, signaling an inflammatory response [96,97]. Overall directionality of the highly expressed DEG suggests an activation of the immune response and enhanced inflammatory response in HE cows.

Research relating RFI to immunity is sparse, and the results are inconclusive. In low- and high-RFI pigs, there was no difference in response when presented with a viral challenge [98], but more feed efficient broiler chickens had increased inflammatory responses and decreased adaptive immune responses [99]. Previous work in early lactation dairy cattle identified copy number variations in genes related to immunity and inflammatory response in the low-RFI cows [100]. The work herein agrees with that of Hou et al. [100], where differences related to immunity and inflammatory response exist between LE and HE cows. Given the glucose utilization of the activated immune system [101], an activated immune response may lead to a reduced feed efficiency; however, the current work is contradictory to that speculation in that some immune response genes were upregulated in HE cows. Given that the cows in this study were not under immune challenge, differences between LE and HE could indicate a state of immune readiness in the absence of full activation. Both immune readiness and activation are reflective of current and long-term energetics in humans [102] and rodents [103]. The novel observations herein warrant future examination with more targeted methodologies to determine the implication of these differences as they contribute to variation in RFI.

#### 4.4.2. Muscle Transcriptome

Despite 163 downregulated DEG and 152 upregulated DEG in the muscle of HE cows, no KEGG pathways were significantly different between HE and LE groups. Enrichment of actin filament binding (GO:0051015) and extracellular space (GO:0005615) in upregulated DEG of HE cows relate to the intercellular structure in the skeletal muscle; conversely, many of the downregulated DEG in HE cows were located within the nucleus (GO:0005634) of the cell. This presents a potential separation of cellular location between upregulated and downregulated DEG between HE and LE cows, which is intriguing.

**Figure 2:**
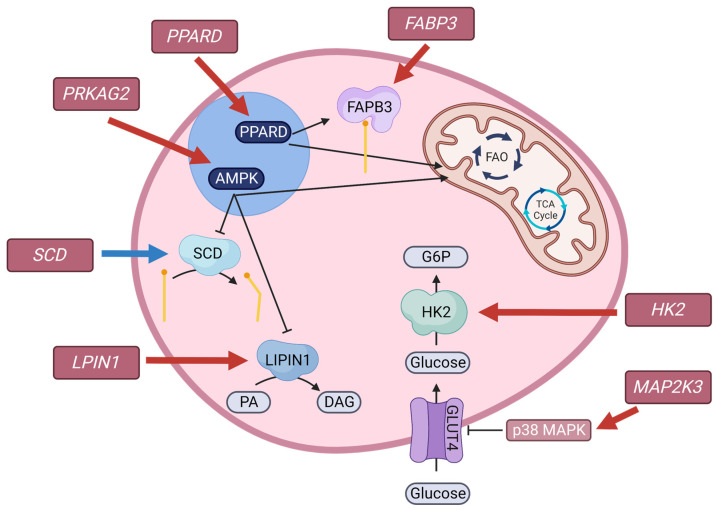
A working hypothesis of differential pathways in skeletal muscle of more feed efficient (HE; *n* = 8) compared with less feed efficient (LE; *n* = 8) cows based on highly expressed differential genes from RNA sequencing. Highly expressed differential genes (rose box; mean read count ≥ 200; *p*-value ≤ 0.05; fold change ≥1.5) that were downregulated (red arrow) or upregulated (blue arrow) are depicted. The *MAP2K3* gene activates the p38 MAPK pathway, which can inhibit glucose transporter 4 (GLUT4) expression, and the *HK2* gene, which encodes hexokinase (HK) is required for the sequestration of glucose. *FABP3* encodes fatty acid binding protein 3 (FABP3), which is required for fatty acid transport. *SCD* encodes stearoyl-CoA desaturase (SCD) and *LPIN1* encodes LIPIN1, which are involved in fatty acid and triglyceride synthesis, respectively. *PRKAG2* encodes a subunit of AMP-activated protein kinase (AMPK) and *PPARD* encodes peroxisome proliferator-activated receptor delta (PPARD), which are responsible for energy sensing and controlling fatty acid oxidation (FAO), respectively. The coordinated downregulation of these genes may be suggestive of reduced oxidation within skeletal muscle of HE cows.

Highly expressed DEG (NMRC > 1000) that were downregulated in HE compared with LE cows were associated with metabolism of glucose (*HK2* and *MAP2K3*), fatty acids (*LPIN1*, *FABP3*, *PPARD*, and *PPARGC1A*), and energy sensing (*PRKAG2*) (Figure 2). Skeletal muscle is an insulin-dependent glucose utilizing tissue, requiring insulin for the translocation of GLUT4, a glucose transporter, to the cell surface. The *MAP2K3* gene encodes mitogen-activated protein kinase 3 and is activated by inflammation, cellular stress, and insulin [104,105]. Activation signals the p38 mitogen-activated protein kinase pathways, leading to a downregulation of GLUT4 expression [105]. Once glucose enters the cell through a glucose transporter, it must be phosphorylated via hexokinase, encoded by *HK2*, to glucose-6-phosphate before it can undergo further metabolism. Downregulation of *HK2* may be suggestive of decreased sequestration of glucose within skeletal muscle. Plasma glucose concentrations between HE and LE cows were not different. A difference in glucose utilization by skeletal muscle with maintained circulating glucose concentrations could reflect differences in liver gluconeogenesis, or differences in the utilization of glucose by other tissues, potentially reflecting a shift in nutrient partitioning to spare glucose for mammary lactose synthesis.

In addition to genes related to glucose metabolism, genes related to lipid metabolism were downregulated in HE cows compared with LE cows. A highly expressed upregulated gene, *SCD*, and a highly expressed downregulated gene, *LPIN1*, encode proteins that are involved in fatty acid and triglyceride synthesis, respectively [106,107]. The gene encoding fatty acid binding protein, *FABP3*, is involved in the cellular uptake of fatty acids and intracellular transport of fatty acids for oxidation [108], and was downregulated in HE cows. Peroxisome proliferator-activated receptors (PPAR) are nuclear receptors that play a role in glucose and fatty acid metabolism, inflammation, and cell fate [109]. Peroxisome proliferator-activated receptor gamma coactivator 1 alpha, encoded by *PPARGC1A*, is regulated by sirtuin 1 in humans and mice [110,111], and may be regulated similarly in bovine [112]. Interestingly, previous work in bovine has indicated that *PPARGC1A* gene expression was affected by circulating fatty acid profile during the transition period in the liver [112]; however, to our knowledge, this is the first study investigating *PPARGC1A* in muscle tissue. Reduction of *PPARGC1A* in muscle may indicate an increase in glucose sequestration and complete oxidation of fatty acids [110,111]. Peroxisome proliferator-activated receptor delta, encoded by *PPARD*, regulates genes related to fatty acid metabolism, including *FABP3*, stimulating fatty acid oxidation [113]. Taken together, the muscle DEG suggest decreased fatty acid oxidation in muscle tissue of HE compared with LE. Interestingly, our previous research indicated more complete oxidation of fatty acids in HE, as measured by circulating plasma acylcarnitines, but that study was based on the presence of intermediates in circulation and was unable to attribute the difference in oxidation to a specific tissue [10]. Given that PPARGC1A and PPARD regulates key genes related to fatty acid oxidation and the generation of acylcarnitines, it is possible that skeletal muscle could be a site of differential oxidation of fatty acids between RFI groups.

Although serum insulin and plasma glucose concentrations were not different by RFI group, differences in genes related to lipid and glucose metabolism suggest possible differences in nutrient partitioning or nutrient requirements of skeletal muscle. Differences in nutrient partitioning or requirements may be, in part, due to cellular energy sensing. AMP-activated protein kinase (AMPK) acts as an energy control switch, in which activation redirects metabolism towards catabolism and against anabolism, serving as a mediator of cellular energy by directly and indirectly controlling metabolism of glucose and lipids [114]. The *PRKAG2* gene encodes one of the three γ-subunits of AMPK and was downregulated in HE cows. Differences in *PRKAG2* suggest a potential role of AMPK in RFI variance and should be further investigated.

## 5. Conclusions

Post-absorptive nutrient metabolism contributes to variation in RFI of dairy cows, but the tissues, and pathways within the tissues, that are altered in more feed efficient cows are unknown. The current work identified novel insights into specific differences in the liver and muscle transcriptome between high (HE) and low (LE) feed efficient animals that represent sources of variation in RFI. Upregulation of the pathways related to the life cycle of the cell in liver tissue, which are tightly related to oxidative stress, and downregulation of DEG related to antioxidants, are suggestive of increased cell cycling and reduced oxidative stress in high feed efficient cows. Several of the highly expressed upregulated DEG in liver were related to adaptive and innate immune response, suggesting altered immune status within the livers of high feed efficient cows. Although KEGG pathways were not enriched in DEG of muscle tissue, highly expressed DEG were involved in the metabolism of glucose and fatty acids, indicating differences between HE and LE cows independent of the differences observed in the liver. Combined, these results suggest that both liver and muscle tissue contribute to sources of variation in RFI, but in different ways. Understanding potential interactions between feed efficiency and nutrition strategies could guide future nutritional approaches. Supplementing 13.0 g/d of choline ion during mid-lactation did not improve productive performance but may impact metabolism differently depending on the feed efficiency status of the mid-lactation dairy cow. Future work should augment the current work by further investigating the interaction between choline supplementation and feed efficiency on metabolism and by determining the oxidative capacity and nutrient use efficiency of mitochondria within the liver and muscle in divergent RFI groups.

## Figures and Tables

**Table 1:** Calculated ingredient composition and nutrient analysis of mid-lactation diets for cows supplemented without (CTL) or with rumen-protected choline (RPC).

	CTL		RPC	
Item, %DM	Mean	SD	Mean	SD
Ingredient composition				
Alfalfa haylage	23.62	0.73	23.87	1.05
Corn silage	29.92	0.97	30.03	1.25
Distillers grain	1.22	0.06	1.18	0.03
Cottonseed	5.09	0.49	5.04	0.37
High moisture corn	12.66	0.37	12.42	0.73
Protein concentrate mix ^1^	27.49	0.62	27.46	0.70
Nutrient analysis				
DM, % as fed	50.04		49.94	
CP	18.10		18.16	
aNDF	28.07		27.65	
aNDFom	27.28		26.85	
ADF	19.98		20.07	
Lignin	3.40		3.37	
NFC	43.73		43.97	
Starch	25.66		25.79	
Fat	4.55		4.52	
NE_L_ 3×, Mcal/kg DM ^2^	1.71		1.71	

^1^ Protein concentrate mix was formulated to contain on a an as-fed basis (CTL, RPC) fine ground corn (25.06%, 25.05%), canola meal (19.92%, 19.91%), soy hull pellet (17.54%, 17.41%), Soy Plus (11.15%, 11.15%; Landus Cooperative, Ames, IA, USA), 46% CP soybean meal (15.66%, 15.66%), calcium carbonate (4.11%, 4.11%), sodium bicarbonate (2.48%, 2.48%), trace mineral salt (1.25%, 1.25%), grease (0.25% 0.25%; Sanimax, Green Bay, WI, USA), magnesium oxide (0.75%, 0.75%), urea (0.63%, 0.63%), potassium carbonate (0.30%, 0.30%), Celmenax dry (0.30%, 0.30%; Arm & Hammer, Princeton, NJ, USA), DynaMate (0.15%, 0.15%; The Mosaic Company, Plymouth, MN, USA), Smartamine M (0.15%, 0.15%; Adisseo, Alpharetta, GA, USA), Fortress LG (0.15%, 0.15%; VitaPlus, Madison, WI, USA), soybean oil (0.13%, 0.0%), and rumen-protected choline prototype (0.0%, 0.30%; Balchem Corp., New Hampton, NY, USA). ^2^ Estimated from NASEM [21] equations to calculate NE_L_ at 3× maintenance.

**Table 2:** Least squares means and 95% confidence intervals of performance variables of low (feed efficient, HE; *n* = 12) and high (feed inefficient, LE; *n* = 12) residual feed intake (RFI) mid-lactation dairy cows.

Variable ^1^	HE		LE		*p*-Value
RFI, kg	−1.73	[−2.16, −1.30]	1.72	[1.17, 2.27]	-
DMI, kg	29.5	[28.5, 30.6]	33.5	[32.5, 34.6]	<0.01
Milk Production					
Milk, kg	50.7	[46.7, 54.8]	51.2	[47.2, 55.2]	0.87
Milk energy, Mcal	33.4	[30.9, 36.0]	34.4	[31.8, 37.0]	0.59
ECM, kg	49.7	[46.0, 53.3]	51.0	[47.3, 54.6]	0.61
FCM, kg	48.4	[44.7, 52.2]	50.0	[46.3, 53.7]	0.54
Fat, kg	2.7	[2.2, 3.2]	3.0	[2.5, 3.5]	0.32
Protein, kg	1.6	[1.4, 1.7]	1.6	[1.4, 1.7]	0.94
Lactose, kg	2.4	[2.2, 2.6]	2.4	[2.2, 2.6]	0.92
Fat, %	3.20	[2.95, 3.45]	3.37	[3.12, 3.62]	0.32
Protein, %	3.06	[2.94, 3.17]	3.06	[2.94, 3.17]	0.98
MUN, mg/dL	14.6	[13.7, 15.6]	15.3	[14.4, 16.2]	0.32
Body Size					
BW, kg	768	[733, 802]	784	[750, 819]	0.49
Metabolic BW	146	[141, 151]	148	[143, 153]	0.50
BW change, kg/d	0.32	[0.12, 0.53]	0.26	[0.06, 0.47]	0.69
BCS	3.22	[3.07, 3.37]	3.15	[3.00, 3.30]	0.48

^1^ DMI = dry matter intake; ECM = energy-corrected milk; FCM = fat-corrected milk; MUN = milk urea nitrogen; BW = body weight; BCS = body condition score.

**Table 3:** Least squares means and 95% confidence intervals of performance variables of mid-lactation cows supplemented without (CTL; *n* = 30) or with rumen-protected choline (RPC; *n* = 31).

Variable ^1^	CTL		RPC		*p*-Value
RFI, kg	−0.05	[−0.53, 0.43]	0.05	[−0.42, 0.52]	0.76
DMI, kg	31.5	[30.7, 32.3]	31.1	[30.3, 31.9]	0.47
Milk Production					
Milk, kg	51.9	[49.6, 54.1]	50.1	[47.9, 52.2]	0.25
Milk energy, Mcal	34.0	[32.6, 35.4]	33.6	[32.2, 35.0]	0.65
ECM, kg	50.6	[48.6, 52.6]	49.8	[47.8, 51.8]	0.58
FCM, kg	49.5	[47.4, 51.6]	48.6	[46.5, 50.7]	0.56
Fat, kg	1.66	[1.57, 1.76]	1.66	[1.57, 1.75]	0.95
Protein, kg	1.57	[1.51, 1.64]	1.56	[1.50, 1.62]	0.72
Lactose, kg	2.46	[2.35, 2.57]	2.38	[2.27, 2.49]	0.30
Fat, %	3.27	[3.10, 3.43]	3.40	[3.24, 3.54]	0.26
Protein, %	3.02	[2.95, 3.08]	3.09	[3.03, 3.17]	0.11
MUN, mg/dL	14.1	[13.6, 14.5]	14.7	[14.2, 15.2]	0.07
Body Size					
BW, kg	778	[755, 802]	759	[736, 782]	0.24
Metabolic BW	0.21	[0.07, 0.36]	0.14	[0.00, 0.28]	0.45
BCS	3.18	[3.04, 3.31]	3.13	[3.00, 3.26]	0.66

^1^ DMI = dry matter intake; ECM = energy-corrected milk; FCM = fat-corrected milk; MUN = milk urea nitrogen; BW = body weight; BCS = body condition score.

**Table 4:** Least squares means and 95% confidence intervals of blood metabolites between low (high feed efficient, HE) and high RFI (low feed efficient, LE) and supplemented with rumen-protected choline (RPC) or not (CTL) in mid-lactation dairy cows.

	HE ^1^				LE ^1^				*p*-Value ^2^		
Metabolite ^3^	CTL		RPC		CTL		RPC		RFI	TRT	RxT
BHB, mmol/L	0.67	[0.54, 0.83]	0.75	[0.60, 0.93]	0.56	[0.45, 0.70]	0.62	[0.50, 0.77]	0.04	0.24	0.97
Glucose, mg/dL	63.3 ^ab^	[60.6, 66.0]	63.9 ^ab^	[61.2, 66.6]	62.8 ^b^	[60.1, 65.6]	67.8 ^a^	[65.1, 70.4]	0.19	0.03	0.09
Fatty acids, mEq/L	0.13	[0.11, 0.16]	0.12	[0.09, 0.14]	0.14	[0.12, 0.17]	0.14	[0.11, 0.16]	0.21	0.38	0.54
Triglyceride, mg/dL	9.9 ^a^	[8.1, 11.7]	7.5 ^b^	[5.7, 9.4]	9.4 ^ab^	[7.1, 11.7]	9.6 ^ab^	[7.4, 11.8]	0.25	0.12	0.05
Albumin, g/dL	3.73	[3.62, 3.86]	3.79	[3.67, 3.93]	3.67	[3.57, 3.78]	3.89	[3.75, 4.06]	0.84	0.03	0.20
Bilirubin, mg/dL	0.09	[0.06, 0.12]	0.11	[0.04, 0.18]	0.10	[0.06, 0.14]	0.08	[0.07, 0.09]	0.66	0.95	0.34
BUN, mg/dL	17.1	[13.9, 20.3]	17.8	[14.6, 21.0]	17.5	[14.6, 20.4]	18.3	[15.5, 21.1]	0.62	0.45	0.96
Creatinine, mg/dL	0.71	[0.64, 0.78]	0.69	[0.62, 0.76]	0.66	[0.59, 0.73]	0.71	[0.64, 0.78]	0.67	0.63	0.35
ALT, U/L	31.4 ^ab^	[27.7, 35.1]	24.7 ^b^	[21, 28.4]	33.0 ^a^	[29.3, 36.7]	36.0 ^a^	[32.3, 39.6]	<0.01	0.31	0.01
AST, U/L	121	[99, 143]	126	[103, 148]	113	[91, 136]	133	[111, 155]	0.98	0.23	0.45
Insulin, µg/L	0.45	[0.30, 0.69]	0.60	[0.39, 0.91]	0.38	[0.25, 0.58]	0.38	[0.25, 0.58]	0.13	0.50	0.49
RQUICKI ^4^	0.53	[0.48, 0.59]	0.54	[0.40, 0.68]	0.56	[0.50, 0.62]	0.54	[0.48, 0.61]	0.67	0.89	0.79

^1^ HE-CTL, *n* = 6; HE-RPC, *n* = 6; LE-CTL, *n* = 6; LE-RPC, *n* = 6. ^2^ RFI= Residual feed intake group; TRT = dietary treatment; RxT = interaction between RFI and TRT. ^3^ BHB = β-hydroxybutyrate; BUN = blood urea nitrogen; ALT = alanine aminotransferase; AST = aspartate aminotransferase. ^4^ Revised quantitative insulin sensitivity check index [30] calculated as: RQUICKI = 1/[log (glucose mg/dL) + log (insulin µIU/mL) + log (non-esterified fatty acids mmol/L)]. ^a,b^ Means without common letters within the same row differed significantly (*p* ≤ 0.05).

**Table 5:** Least squares means and 95% confidence intervals of blood plasma fatty acids between low (high feed efficient, HE) and high (low feed efficient, LE) and supplemented with rumen-protected choline (RPC) or not (CTL) in mid-lactation dairy cows.

	HE ^1^				LE ^1^				*p*-Value ^2^		
Fatty Acid, mg/dL ^3^	CTL		RPC		CTL		RPC		RFI	TRT	RxT
C14:0	0.42	[0.38, 0.47]	0.46	[0.42, 0.51]	0.46	[0.42, 0.50]	0.43	[0.38, 0.47]	0.95	0.81	0.08
C15:0	0.31	[0.28, 0.33]	0.31	[0.28, 0.33]	0.32	[0.29, 0.34]	0.32	[0.29, 0.34]	0.31	0.94	0.94
C16:0	4.10	[3.63, 4.56]	4.01	[3.55, 4.47]	4.00	[3.53, 4.46]	4.20	[3.74, 4.67]	0.84	0.79	0.52
C16:1	0.69	[0.61, 1.03]	0.64	[0.63, 0.69]	0.67	[0.63, 0.73]	0.67	[0.62, 0.74]	0.85	0.91	0.17
C17:0	0.87	[0.78, 0.98]	0.73	[0.66, 0.80]	0.80	[0.72, 0.89]	0.87	[0.78, 0.99]	0.29	0.36	0.02
C18:0	4.72	[4.16, 5.45]	4.52	[4.01, 5.19]	4.64	[4.10, 5.35]	4.93	[4.32, 5.73]	0.59	0.91	0.43
C18:1	3.15	[2.84, 3.47]	3.32	[3.00, 3.63]	3.35	[3.03, 3.66]	3.14	[2.83, 3.46]	0.94	0.90	0.24
C18:2	13.87	[11.8, 15.9]	12.18	[10.1, 14.2]	13.63	[11.5, 15.7]	14.71	[12.7, 16.8]	0.24	0.75	0.16
C18:3	2.21	[1.88, 2.53]	2.10	[1.78, 2.43]	2.30	[1.98, 2.63]	2.35	[2.03, 2.67]	0.28	0.86	0.63
C20:3	1.39	[1.33, 1.45]	1.36	[1.30, 1.41]	1.40	[1.25, 1.54]	1.44	[1.30, 1.59]	0.38	0.91	0.43
C20:4	0.73	[0.69, 0.76]	0.78	[0.74, 0.82]	0.73	[0.70, 0.77]	0.74	[0.71, 0.78]	0.37	0.09	0.21

^1^ HE-CTL, *n* = 6; HE-RPC, *n* = 6; LE-CTL, *n* = 6; LE-RPC, *n* = 6. ^2^ RFI= Residual feed intake group; TRT = dietary treatment; RxT = interaction between RFI and TRT. ^3^ C16:1 = myristoleate (*cis*-9); C18:1 = oleate (*cis*-9); C18:2 = linoleate (*cis*-9,12); C18:3 = linolenate (*cis*- 9,12,15); C20:3 = eicosatrienoate (*cis*-11,14,17); C20:4 = arachidonic acid (*cis*-5,8,11,14).

**Table 6:** Kyoto Encyclopedia of Genes and Genomes (KEGG) metabolic pathways enriched in upregulated differentially expressed genes in liver samples from cows that were high feed efficient (HE; *n* = 12) compared with low feed efficient (LE; *n* = 12) ^1^.

KEGG Pathway	ID	Genes	Fold Enrichment	Corrected *p*-Value	Gene Symbols ^2^
Cell cycle	bta04110	14	8.4	6.9 ×10^−12^	*BUB1*, *BUB1B*, *CCNA2*, *CCNB1*, *CCNB2*, *CCNE2*, *CDC20*, *CDC25A*, *CDC6*, *CDK1*, *CDKN2C*, *ESPL1*, *ORC1*, *TTK*
Cellular senescence	bta04218	8	4.8	0.00067	*CCNA2*, *CCNB1*, *CCNB2*, *CCNE2*, *CDC25A*, *CDK1*, *FOXM1*, *LOC787122*
Human T-cell leukemia virus 1 infection	bta05166	8	4.8	0.00540	*BUB1B*, *CCNA2*, *CCNB2*, *CCNE2*, *CDC20*, *CDKN2C*, *ESPL1*, *LOC787122*
p53 signaling pathway	bta04115	5	3.0	0.01500	*CCNB1*, *CCNB2*, *CCNE2*, *CDK1*, *RRM2*

^1^ Enrichment analysis was performed using the Database for Annotation, Visualization, and Integrated Discovery (v. 2021) by comparing differentially expressed genes (mean read count ≥ 10; *p*-value ≤ 0.05; fold change ≥1.5). Fold enrichment and Benjamini corrected *p*-values are reported. ^2^ Annotation of gene transcripts and affiliated gene symbols are based on the *Bos taurus* reference genome (release 106, ARS-UCS 1.2).

**Table 7:** Gene Ontology domains enriched in differentially expressed genes in muscle samples from cows that were high feed efficient (HE; *n* = 8) compared with low feed efficient (*n* = 8) ^1^.

Gene Ontology Domain	ID	Genes	Fold Enrichment	Corrected *p*-Value
Upregulated in HE				
Cell Component: extracellular space	GO:0005615	19	2.9	0.0130
Molecular Function: actin filament binding	GO:0051015	8	5.3	0.0140
Downregulated in HE				
Cell Component: nucleus	GO:0005634	44	1.9	0.0061

^1^ Enrichment analysis was performed using the Database for Annotation, Visualization, and Integrated Discovery (v. 2021) by comparing differentially expressed genes (mean read count ≥ 10; *p*-value ≤ 0.05; fold change ≥1.5). Fold enrichment and Benjamini corrected *p*-values are reported.

## Data Availability

Feed efficiency data were collected under a specific collaborative agreement to provide data for national genetic evaluation of U.S. dairy cattle for Residual Feed Intake and Feed Saved. These data are available on request of the corresponding author and approval by the collaborators, assuming that the purpose is not to duplicate the objectives of the national collaborative project.

## Supplementary Materials

The following supporting information can be downloaded at: https://doi.org/10.5281/zenodo.8200748, Supplemental Materials; Table S1: Least squares means and 95% confidence intervals of blood metabolites between mid-lactation cows supplemented with rumen-protected choline (RPC) or not (CTL); Table S2: Least squares means and 95% confidence intervals of blood plasma fatty acids between mid-lactation cows supplemented with rumen-protected choline (RPC) or not (CTL); Table S3: Differentially expressed genes in the liver tissue between feed efficient (HE) and feed inefficient (LE) mid-lactation cows ^1^; Table S4: Gene Ontology domains enriched in upregulated differentially expressed genes in liver samples from cows that were high feed efficient (HE; *n* = 12) or low feed efficient (LE; *n* = 12) ^1^; Table S5: Differentially expressed genes in muscle tissue between high feed efficient (HE) and low feed efficient (LE) mid-lactation cows ^1^; Table S6: Gene Ontology domains enriched in differentially expressed genes in muscle samples from cows that were high feed efficient (HE; *n* = 8) or low feed efficient (LE; *n* = 8) ^1^.
