# Supplementary material for: Liver and Muscle Transcriptomes Differ in Mid-Lactation Cows Divergent in Feed Efficiency in the Presence or Absence of Supplemental Rumen-Protected Choline
Source: Metabolites. 2023 Sep 19;13(9):1023. doi: 10.3390/metabo13091023 (PMC10536747; doi:10.3390/metabo13091023)
Supplement: Supplementary file 1 [file metabolites-13-01023-s001.zip › metabolites-2607995-supplementary.pdf]

SUPPLEMENTARY MATERIALS

Midpoint Residual Feed Intake Calculation

To reduce the number of cows subjected to biopsy procedures, liver and muscle tissue samples were collected by biopsy from 32 of the 64 cows. The 32 cows selected for biopsies were identified by a midpoint RFI calculation. Dry matter intake for each cow was computed as a function of major energy sinks using the lm function in R 4.2.1 (R Core Team, 2022). The midpoint RFI model was:

$$DMI = \mu + MilkE + \beta 2 \times MBW + \beta 3 \times \Delta BW + \beta 4 \times DIM + RFI,$$

where DMI was the observed DMI (kg), μ is the overall mean, and MilkE, MBW, ΔBW, and DIM are the secreted milk energy, metabolic BW, daily change in BW, and DIM, respectively. Regression coefficients β1, β2, β3, and β4 correspond to secreted milk energy, metabolic BW, daily change in BW, and DIM, respectively. The random residual, RFI, was the RFI phenotype measured. The 16 cows with the highest RFI and the 16 cows with the lowest RFI, balanced by dietary treatment, were selected for biopsies, with the goal of capturing the true top 12 and bottom 12 cows when calculating the final RFI.

Between the midpoint and final RFI calculations, minor re-ranking occurred (spearman's correlation coefficient = 0.91). However, the true top 12 and bottom 12 of RFI were maintained in the top 13 and bottom 12, respectively, in the final RFI.

Blood Metabolite Quantification

Quantification of plasma BHB (C444-OA, Catachem), glucose (C124-06, Catachem), albumin (C244-01, Catachem), BUN (C264-03, Catachem), creatinine (V320-24, Catachem),

alanine aminotransferase (**ALT**; C164-0A, Catachem) , and aspartate amino transferase (**AST**; C164-0A, Catachem) were based on previously described chemistries and determined using Catachem VETSPEC reagents on the Catachem Well-T AutoAnalyzer using recommended protocols from the manufacturer (Catachem, Oxford, CT). A linear standard curve was generated using standards from the manufacturer suitable for each metabolite (C444-10, C444-11, and C444-12 for BHB; C1200-10, C1200-11, and C1200-12 for albumin, BUN, creatinine, glucose, AST, and ALT; Catachem). Standards were within the expected calibrated ranges provided by the manufacturer (Catachem). Samples were read by the autoanalyzer in cuvettes either in duplicate (BHB, glucose, albumin, BUN) or triplicate (ALT and AST). A plasma pool was run with every set of samples. The concentration or activity of the unknown samples were determined based on the linear standard curve.

The concentration of BHB was determined using BHB dehydrogenase to measure the reduction of NAD to NADH and read at 340 nm (Koch and Feldbruegge, 1987). Concentration of glucose was determined from the NADH produced from using hexokinase to generate glucose-6-phosphate and subsequent oxidation to phophoglucono-lactone and read at 340 nm (León et al., 1977). Albumin concentration was determined using tetrabromo-m-cresolphthalein dye and read at 630 nm (Doumas et al., 1971). Concentration of BUN was determined using urease and glutamate dehydrogenase to measure the oxidation of acetyl-NADH to acetyl-NAD and read at 340 nm (Talke and Schubert, 1965). Creatinine concentration was determined using picric acid to produce a red Tautomer complex and read at 505 nm (Chasson et al., 1960). Activity of ALT was determined using L-alanine and α-ketoglutarate to measure the oxidation of NADH+ to NAD from pyruvate and read at 340 nm (Provisional recommendations on IFCC methods for the measurement of catalytic concentrations of enzymes. Part 2. IFCC method for aspartate aminotransferase, 1977).

Activity of AST was determined using L-aspartate and α-ketoglutarate to measure the oxidation of NADH+ to NAD from oxaloacetate and read at 340 nm (Bergmeyer et al., 1976).

Quantification of plasma triglyceride (C116-0A, Catachem) was quantified using Catachem VETSPEC reagents using a modified protocol. Determination of triglyceride concentration was based on a multi-step reaction in which final product, quinonemine dye, was read at 505 nm (Trinder, 1969). In place of using the Catachem specific standards (C1200-10, C1200-11, and C1200-12; Catachem) to generate the linear standard curve, a serial dilution (1:2) of a standard (464-01601; Fujifilm Wako Chemicals USA) was used to establish the linear standard curve. Samples were quantified using the generated standard curve in place of the auto-analyzer generated curve.

Quantification of plasma fatty acids (C514-0A; Catachem) and serum bilirubin (C304-0A; Catachem) were quantified using Catachem VETSPEC reagents with modified protocols and read on a Synergy H1 Hybrid Spectrophotometer (BioTek, Winooski, VT, USA). A serial dilution (1:2) of a standard for fatty acids (NEFA standard solution; Fujifilm Wako Chemicals USA) and for bilirubin (C1200-10; Catachem) were used to establish a standard curve for the quantification of fatty acids and bilirubin, respectively. The concentration of fatty acids was determined using a multi-step reaction in which the final product, quinoneindamine dye, was read at 545 nm (Trout et al., 1960; Itaya and Ui, 1965; Novak, 1965). The concentration of bilirubin was determined using diazotized sulfanilic acid to form an azo-Bilirubin complex and read at 545 nm (Van den Bergh and Snapper, 1913).

Muscle Tissue Biopsy

Semitendinosus muscle tissue were collected from the right upper hindlimb. The right upper hindlimb region was cleaned from debris using a stiff brush, shaved, and the tail tied off to the left using hosiery. The surgical field was prepared by alternating washes of povidone iodine (0.75% titratable iodine, First Priority, Elgin, IL) and 70% ethanol solutional, 3 times each. A 10 mL injection of lidocaine hydrochloride injectible-2% (Clipper Distribution Company, St. Joseph, MO) was given subcutaneously at the planned incision site. A straight-line incision was made (~ 1 inch) and a sterile 8 mm punch biopsy tool (Miltex model 33-57, Integra LifeSciences, Princeton, NJ) was used to collect muscle tissue (~ 1 g). The incision was sutured with sterile non-absorbable suture material (USP 1, Braunamid white, Jorgensen Lab, Loveland, CO). The incision site and health of the cow was monitored by the research staff and a veterinarian for 5 d post operation. Sutures were removed at 7 to 10 d post operation.

REFERENCES

Van den Bergh, A.A.H., and J. Snapper. 1913. Die Farbstoffe des Blutserums. Dtsch. Arch. Klin. Med. 110:540.

Bergmeyer, H.U., G.N.J. Bowers, M. Horder, and D.W. Moss. 1976. Provisional recommendations on IFCC methods for the measurement of catalytic concentrations of enzymes. Part 2. IFCC method for aspartate aminotransferase. Clin. Chim. Acta. 70:F19-29.

Chasson, A.L., H.J. Grady, and M.A. Stanley. 1960. Determination of creatinine by means of automatic chemical analysis. Tech. Bull. Regist. Med. Technol. Am. Soc. Clin. Pathol. Regist. Med. Technol. 30:207–212.

Doumas, B.T., W.A. Watson, and H.G. Biggs. 1971. Albumin standards and the measurement of serum albumin with bromcresol green. Clin. Chim. Acta. 31:87–96. https://doi.org/10.1016/0009-8981(71)90365-2.

Holtenius, P., and K. Holtenius. 2007. A model to estimate insulin sensitivity in dairy cows. Acta Vet. Scand. 49. https://doi.org/10.1186/1751-0147-49-29.

Itaya, K., and M. Ui. 1965. Colormetric determination of free fatty acids in biological fluids. J. Lipid Res. 6:16–20.

Koch, D.D., and D.H. Feldbruegge. 1987. Optimized kinetic method for automated determination of β-hydroxybutyrate. Clin. Chem. 33:1761–1766. https://doi.org/10.1093/clinchem/33.10.1761.

León, L.P., M. Sansur, L.R. Snyder, and C. Horvath. 1977. Continuous-flow analysis for glucose, triglycerides, and ATP with immobilized enzymes in tubular form. Clin. Chem. 23:1556–1562

Novak, M. 1965. Colormetric ultramicro method for the determination of free fatty acids. J. Lipid Res. 6:431–433.

Provisional recommendations on IFCC methods for the measurement of catalytic concentrations of enzymes. Part 2. IFCC method for aspartate aminotransferase. 1977. . J. Clin. Chem. Clin. Biochem. 15:39–51.

Talke, H., and G.E. Schubert. 1965. Enzymatic urea determination in the blood and serum in the Warbug optical test. Klin. Wochenschr. 43:174–175. https://doi.org/10.1007/BF01484513.

Trinder, P. 1969. Determination of glucose in blood using glucose oxidate with an alternative oxygen acceptor. Ann. Clin. Biochem. 6:24. https://doi.org/https://doi.org/10.1177/000456326900600108.

Trout, D.L., E.H.J. Estes, and S.J. Friedberg. 1960. Titration of free fatty acids of plasma: a study of current methods and a new modification. J. Lipid Res. 1:199–202.

Supplemental Table S1. Least squares means and 95% confidence intervals of blood metabolites between mid-lactation cows supplemented with rumen protected choline (RPC) or not (CTL).

Metabolite[2]	Treatment[1]				*P*-value
	CTL		RPC		
BHB, mmol/L	0.67	[0.57, 0.78]	0.68	[0.58, 0.79]	0.83
Glucose, mg/dL	62.4	[60.6, 64.2]	63.6	[61.9, 65.3]	0.29
Fatty acids, mmol/L	0.13	[0.12, 0.15]	0.13	[0.11, 0.14]	0.45
Triglyceride, mg/dL	9.0	[8.1, 9.9]	8.5	[7.6, 9.4]	0.25
Albumin, g/dL	3.73	[3.69, 3.78]	3.82	[3.75, 3.89]	0.05
Bilirubin, mg/dL	0.08	[0.07, 0.11]	0.08	[0.06, 0.10]	0.31
BUN, mg/dL	17.5	[19.2, 16]	17.7	[19.4, 16.2]	0.73
Creatinine, mg/dL	0.67	[0.64, 0.71]	0.69	[0.66, 0.73]	0.32
ALT, U/L	32.2	[29.9, 34.6]	30.3	[28.3, 32.5]	0.24
AST, U/L	123	[110, 136]	132	[120, 145]	0.20
Insulin, µg/L	0.46	[0.32, 0.67]	0.48	[0.33, 0.7]	0.77
RQUICKI[3]	0.53	[0.49, 0.57]	0.54	[0.5, 0.58]	0.42

[1]Cows were randomly assigned to treatment. RPC, n = 31; CTL, n = 29. The PROC MIXED procedures of SAS (v 9.4, SAS Institute Inc., Cary, NC) were used and the model included the fixed effect of treatment and the random effect of day of sampling. Non-normal residuals resulted in the data being transformed via Box-Cox transformation. Persistent non-normal residuals were subject to modeling heterogeneous variance using PROC GLIMMIX.

[2]ALT = alanine aminotransferase; AST = aspartate aminotransferase.

[3]Revised quantitative insulin sensitivity check index (Holtenius and Holtenius, 2007) calculated as: RQUICKI = 1/[log (glucose mg/dL) + log (insulin µIU/mL) + log (non-esterified fatty acids mmol/L)].

Supplemental Table S2. Least squares means and 95% confidence intervals of blood plasma fatty acids between mid-lactation cows supplemented with rumen protected choline (RPC) or not (CTL).

Fatty Acid, mg/dL[2]	Treatment[1]				P-value
	CTL		RPC		
C14:0	0.43	[0.41, 0.45]	0.44	[0.42, 0.46]	0.92
C15:0	0.31	[0.29, 0.32]	0.31	[0.29, 0.32]	0.92
C16:0	3.94	[3.71, 4.18]	3.97	[3.74, 4.2]	0.83
C16:1	0.65	[0.63, 0.68]	0.65	[0.63, 0.68]	0.94
C17:0	0.80	[0.75, 0.86]	0.80	[0.75, 0.85]	0.91
C18:0	4.60	[4.25, 4.95]	4.61	[4.26, 4.95]	0.98
C18:1	3.15	[2.91, 3.38]	3.16	[2.96, 3.37]	0.89
C18:2	13.21	[12.11, 14.31]	13.10	[12.02, 14.17]	0.85
C18:3	2.20	[2.04, 2.35]	2.21	[2.06, 2.36]	0.88
C20:3	1.33	[1.26, 1.40]	1.36	[1.29, 1.42]	0.56
C20:4	0.74	[0.72, 0.76]	0.74	[0.73, 0.76]	0.76

[1]Cows were randomly assigned to treatment. RPC, n = 31; CTL, n = 29. The PROC MIXED procedures of SAS (v 9.4, SAS Institute Inc., Cary, NC) were used and the model included the fixed effect of treatment and the random effect of day of sampling. Non-normal residuals resulted in the data being transformed via Box-Cox transformation. Persistent non-normal residuals were subject to modeling heterogeneous variance using PROC GLIMMIX.
[2]C16:1 = myristoleate (cis-9); C18:1 = oleate (cis-9); C18:2 = linoleate (cis-9,12); C18:3 = linolenate (cis-9,12,15); C20:3 = eicosatrienoate (cis-11,14,17); C20:4 = arachidonate (cis-5,8,11,14).

Supplemental Table S3. Differentially expressed genes in the liver tissue between feed efficient (HE) and feed inefficient (LE) mid-lactation cows[1].

Gene[2]	Gene Symbol	Mean[3]	Fold Change	*P*-value	Expression[4]
acetyl-CoA carboxylase beta	ACACB	143	2.20	0.00066	Down
ADAM metallopeptidase domain 22	ADAM22	318	1.65	0.00197	Up
AHNAK nucleoprotein 2	AHNAK2	79	1.71	0.03924	Down
anillin, actin binding protein	ANLN	172	1.61	0.00860	Up
acyloxyacyl hydrolase	AOAH	397	1.74	0.00146	Up
adaptor related protein complex 3 subunit mu 2	AP3M2	65	1.60	0.00459	Down
apolipoprotein B mRNA editing enzyme catalytic subunit 2	APOBEC2	69	1.90	0.00365	Down
aquaporin 2	AQP2	17	3.19	0.00247	Down
Rho GTPase activating protein 11A	ARHGAP11A	101	1.95	0.00662	Up
assembly factor for spindle microtubules	ASPM	174	1.69	0.02518	Up
basic helix-loop-helix family member a15	BHLHA15	303	1.62	0.02782	Down
basic helix-loop-helix family member e23	BHLHE23	12	1.89	0.00551	Down
baculoviral IAP repeat containing 5	BIRC5	89	1.73	0.01176	Up
BUB1 mitotic checkpoint serine/threonine kinase	BUB1	104	2.10	0.00123	Up
BUB1 mitotic checkpoint serine/threonine kinase B	BUB1B	94	2.15	0.00152	Up
chromosome 16 C1orf105 homolog	C16H1orf105	97	1.52	0.01098	Up
uncharacterized C20H5orf49	C20H5orf49	88	4.29	0.00388	Down
chromosome 2 C2orf88 homolog	C2H2orf88	326	2.43	0.02675	Down
C-C motif chemokine ligand 24	CCL24	76	3.45	0.00585	Down
C-C motif chemokine ligand 26	CCL26	15	1.67	0.01799	Down
cyclin A2	CCNA2	47	1.65	0.04497	Up
cyclin B1	CCNB1	41	1.74	0.03062	Up
cyclin B2	CCNB2	34	1.60	0.02762	Up
cyclin E2	CCNE2	87	1.51	0.00096	Up
cyclin F	CCNF	38	1.59	0.04520	Up
CD180 molecule	CD180	306	1.50	0.02233	Up
CD1e molecule	CD1E	26	1.98	0.00602	Up
CD300e molecule	CD300E	61	1.64	0.02632	Up
cell division cycle 20	CDC20	44	1.65	0.03412	Up
cell division cycle 25A	CDC25A	49	1.61	0.03726	Up
cell division cycle 6	CDC6	35	2.11	0.00287	Up
cell division cycle associated 8	CDCA8	22	1.58	0.04699	Up
cyclin dependent kinase 1	CDK1	142	1.57	0.04411	Up
cyclin dependent kinase inhibitor 2C	CDKN2C	87	1.71	0.00100	Up
centromere protein E	CENPE	125	1.90	0.00517	Up
centromere protein F	CENPF	406	2.07	0.00902	Up
centromere protein W	CENPW	12	2.05	0.00481	Up

centrosomal protein 55	CEP55	30	2.28	0.00328	Up
cilia and flagella associated protein 43	CFAP43	184	1.53	0.00584	Up
cholinergic receptor muscarinic 1	CHRM1	109	1.94	0.01613	Down
circadian associated repressor of transcription	CIART	34	1.56	0.02981	Down
cellular inhibitor of PP2A	CIP2A	146	1.50	0.01235	Up
Cbp/p300 interacting transactivator with Glu/Asp rich carboxy-terminal domain 1	CITED1	10	1.79	0.02429	Down
cytoskeleton associated protein 2	CKAP2	250	1.71	0.00677	Up
cytoskeleton associated protein 2 like	CKAP2L	84	1.91	0.00199	Up
chloride voltage-gated channel 2	CLCN2	237	1.87	0.00000	Down
C-type lectin domain family 4 member E	CLEC4E	21	1.96	0.00313	Up
C-type lectin domain containing 6A	CLEC6A	114	1.82	0.00174	Up
C-type lectin domain containing 7A	CLEC7A	202	1.51	0.00323	Up
claspin	CLSPN	76	1.60	0.01970	Up
carboxypeptidase M	CPM	155	1.53	0.04376	Up
cysteine rich with EGF like domains 2	CRELD2	602	1.88	0.00510	Down
cytochrome P450, family 2, subfamily J	CYP2J2	30	1.53	0.01949	Up
uncharacterized CYR61	CYR61	170	1.60	0.01665	Down
DEP domain containing 1	DEPDC1	46	1.99	0.01920	Up
diaphanous related formin 3	DIAPH3	30	1.81	0.01192	Up
DLG associated protein 5	DLGAP5	69	2.11	0.00270	Up
DNA replication helicase/nuclease 2	DNA2	27	1.67	0.00886	Up
delta/notch like EGF repeat containing	DNER	25	2.19	0.01947	Up
E2F transcription factor 7	E2F7	16	1.71	0.02325	Up
E2F transcription factor 8	E2F8	23	1.98	0.01303	Up
epithelial cell transforming 2	ECT2	110	1.63	0.01574	Up
epithelial membrane protein 1	EMP1	67	1.51	0.03007	Up
ERCC excision repair 6 like, spindle assembly checkpoint helicase	ERCC6L	49	1.77	0.00254	Up
establishment of sister chromatid cohesion N-acetyltransferase 2	ESCO2	62	1.55	0.01277	Up
extra spindle pole bodies like 1, separase	ESPL1	81	1.61	0.04671	Up
exonuclease 1	EXO1	20	2.24	0.00492	Up
uncharacterized FAM19A3	FAM19A3	64	2.41	0.00055	Down
FA complementation group I	FANCI	68	1.72	0.00504	Up
F-box protein 5	FBXO5	46	1.85	0.00164	Up
fibroblast growth factor 21	FGF21	14	3.25	0.04477	Up
forkhead box M1	FOXM1	44	1.56	0.02903	Up
FRAS1 related extracellular matrix 3	FREM3	40	2.48	0.00125	Down
polypeptide N-acetylgalactosaminyltransferase 5	GALNT5	61	2.48	0.03855	Down
growth arrest specific 2 like 3	GAS2L3	141	1.56	0.00083	Up
GTPase, IMAP family member 6	GIMAP6	826	2.28	0.01396	Up
GTPase, IMAP family member 8	GIMAP8	4723	2.88	0.01242	Up
GINS complex subunit 2	GINS2	22	1.65	0.03137	Up

gliomedin	GLDN	111	1.75	0.00382	Up
GDP-mannose pyrophosphorylase B	GMPPB	1393	1.72	0.00715	Down
glycine N-methyltransferase	GNMT	10	3.28	0.01224	Down
glycoprotein V platelet	GP5	47	1.77	0.00037	Down
glutathione peroxidase 3	GPX3	7797	3.28	0.02324	Down
glutathione S-transferase pi 1	GSTP1	790	1.56	0.04274	Down
histone H2B-like	H2B	67	1.51	0.03728	Up
uncharacterized HIST1H1B	HIST1H1B	278	1.84	0.03058	Up
uncharacterized HIST1H2BI	HIST1H2BI	49	1.89	0.00710	Up
uncharacterized HIST1H2BJ	HIST1H2BJ	16	2.35	0.00399	Up
uncharacterized HIST1H2BN	HIST1H2BN	149	1.90	0.01139	Up
uncharacterized HIST1H3C	HIST1H3C	23	1.67	0.04320	Up
uncharacterized HIST1H3G	HIST1H3G	142	1.60	0.02595	Up
uncharacterized HIST2H2BF	HIST2H2BF	140	1.52	0.02146	Up
Holliday junction recognition protein	HJURP	105	1.61	0.03547	Up
hydroxy-delta-5-steroid dehydrogenase, 3 beta- and steroid delta-isomerase 1	HSD3B1	180	1.55	0.01370	Down
hypoxia up-regulated 1	HYOU1	3280	1.81	0.00527	Down
indoleamine 2,3-dioxygenase 2	IDO2	3473	1.59	0.00006	Down
insulin like growth factor 2 mRNA binding protein 3	IGF2BP3	38	2.41	0.01865	Up
interleukin 17B	IL17B	66	2.17	0.00425	Down
potassium voltage-gated channel subfamily H member 7	KCNH7	36	4.40	0.00089	Down
kinesin family member 11	KIF11	124	2.13	0.00172	Up
kinesin family member 15	KIF15	55	2.36	0.00056	Up
kinesin family member 18A	KIF18A	44	1.91	0.00176	Up
kinesin family member 20A	KIF20A	110	1.62	0.03481	Up
kinesin family member 22	KIF22	53	1.72	0.02123	Up
kinesin family member 2C	KIF2C	30	1.70	0.03276	Up
kinesin family member 4A	KIF4A	81	2.10	0.00240	Up
kinesin family member C1	KIFC1	74	1.55	0.03699	Up
killer cell lectin like receptor F1	KLRF1	25	1.70	0.04322	Up
kinetochore scaffold 1	KNL1	182	2.12	0.00087	Up
kinetochore associated 1	KNTC1	107	1.68	0.00582	Up
interleukin 32-like	LOC100139916	59	4.98	0.00368	Down
uncharacterized LOC100196897	LOC100196897	13	1.84	0.00783	Up
WAP four-disulfide core domain protein 18	LOC100296618	22	5.02	0.00859	Up
translation initiation factor IF-2-like	LOC100847999	28	1.53	0.00249	Up
uncharacterized LOC100848294	LOC100848294	20	1.93	0.01584	Up
uncharacterized LOC100848642	LOC100848642	13	1.61	0.01591	Down
uncharacterized LOC100849050	LOC100849050	36	1.53	0.00329	Up
uncharacterized LOC100850437	LOC100850437	49	1.63	0.04618	Down
uncharacterized LOC101901915	LOC101901915	15	1.90	0.00178	Up

uncharacterized LOC101902043	LOC101902043	42	1.78	0.00074	Up
GTPase IMAP family member 7-like	LOC101902675	20	2.57	0.00086	Up
isocitrate dehydrogenase [NADP] cytoplasmic pseudogene	LOC101903193	35	1.58	0.03600	Up
60S ribosomal protein L15 pseudogene	LOC101903301	52	3.74	0.00074	Down
uncharacterized LOC101903832	LOC101903832	64	1.64	0.01128	Down
cationic amino acid transporter 3-like	LOC101904151	24	1.51	0.04679	Up
zinc finger protein 160 pseudogene	LOC101905179	157	1.77	0.00005	Down
fibrous sheath-interacting protein 2-like	LOC101905293	119	1.61	0.03248	Up
heme-binding protein 1 pseudogene	LOC101905801	110	2.60	0.04838	Up
BOLA class I histocompatibility antigen, alpha chain BL3-7-like	LOC101905956	4383	1.50	0.01116	Up
ubiquitin-fold modifier 1 pseudogene	LOC101906021	27	3.17	0.01716	Down
uncharacterized LOC101907141	LOC101907141	19	1.78	0.00613	Down
zinc finger protein 852-like	LOC101909173	107	1.64	0.00108	Down
uncharacterized LOC104968446	LOC104968446	59	2.14	0.00399	Up
uncharacterized LOC104968456	LOC104968456	128	1.98	0.00160	Up
uncharacterized LOC104968518	LOC104968518	16	1.65	0.00093	Up
uncharacterized LOC104969356	LOC104969356	33	1.67	0.04232	Up
uncharacterized LOC104969981	LOC104969981	15	2.06	0.03716	Down
uncharacterized LOC104973604	LOC104973604	12	3.45	0.00483	Up
uncharacterized LOC104975099	LOC104975099	14	1.65	0.00131	Down
uncharacterized LOC104975676	LOC104975676	42	2.09	0.00490	Up
uncharacterized LOC104976195	LOC104976195	53	1.85	0.03385	Down
uncharacterized LOC107132336	LOC107132336	19	1.54	0.01273	Up
uncharacterized LOC107133343	LOC107133343	1171	3.98	0.02606	Down
interferon-induced transmembrane protein 3-like	LOC112441484	505	2.32	0.00729	Down
small nucleolar RNA SNORD67	LOC112441674	50	1.55	0.01922	Up
immunoglobulin lambda-like polypeptide 5	LOC112442062	25	1.83	0.01927	Down
sialic acid-binding Ig-like lectin 14	LOC112442215	257	2.21	0.01502	Up
translation initiation factor IF-2-like	LOC112442408	1315	1.53	0.04211	Down
potassium voltage-gated channel subfamily H member 7-like	LOC112442562	29	4.22	0.00094	Down
uncharacterized LOC112442703	LOC112442703	12	1.95	0.04911	Down
U1 spliceosomal RNA	LOC112442818	37556	3.00	0.03488	Down
uncharacterized LOC112443819	LOC112443819	46	2.08	0.04066	Up
uncharacterized LOC112443858	LOC112443858	14	1.57	0.00935	Down
uncharacterized LOC112444463	LOC112444463	27	3.88	0.00000	Down
uncharacterized LOC112444776	LOC112444776	22	1.50	0.04044	Up
uncharacterized LOC112444778	LOC112444778	24	1.55	0.01634	Up
uncharacterized LOC112444920	LOC112444920	25	1.59	0.02206	Down
endogenous retrovirus group K member 25 Env polyprotein-like	LOC112446744	19	2.07	0.03188	Up
proline-rich protein 2-like	LOC112446784	81	1.67	0.02989	Down
uncharacterized LOC112447393	LOC112447393	62	1.79	0.00634	Down

pleckstrin homology domain-containing family B member 2 pseudogene	LOC112447770	10	1.86	0.00240	Up
uncharacterized LOC112448737	LOC112448737	27	1.55	0.02007	Up
uncharacterized LOC112448796	LOC112448796	22	1.69	0.00238	Up
uncharacterized LOC505183	LOC505183	147	1.72	0.00636	Up
ring finger protein 213-like	LOC512869	330	1.54	0.02054	Down
vascular cell adhesion molecule 1-like	LOC534578	204	2.09	0.03571	Up
nucleosome assembly protein 1-like 1	LOC614785	13	1.59	0.02082	Up
phosphatidylcholine transfer protein	LOC616574	82	1.66	0.00026	Up
BOLA class I histocompatibility antigen, alpha chain BL3-7-like	LOC617979	293	3.55	0.00863	Up
patched domain-containing protein 3	LOC782456	19	1.61	0.00589	Down
ADP/ATP translocase 1	LOC787122	19	2.11	0.00645	Up
uncharacterized LOC787465	LOC787465	99	1.82	0.00692	Up
bax inhibitor 1 pseudogene	LOC788342	39	1.60	0.00616	Up
solute carrier family 22 member 10-like	LOC788786	25	1.73	0.01143	Up
uncharacterized LOC788915	LOC788915	94	4.98	0.04609	Down
sialic acid-binding Ig-like lectin 14	LOC789748	363	2.23	0.02664	Up
leucine rich single-pass membrane protein 2	LSMEM2	27	1.95	0.04434	Up
latexin	LXN	93	1.77	0.00079	Up
lysozyme	LYZ	894	1.73	0.00010	Up
mitogen-activated protein kinase 12	MAPK12	204	1.51	0.00620	Down
macrophage receptor with collagenous structure	MARCO	2813	2.20	0.01908	Up
microtubule associated serine/threonine kinase like	MASTL	44	1.91	0.00141	Up
maternal embryonic leucine zipper kinase	MELK	40	1.71	0.03801	Up
MIS18 binding protein 1	MIS18BP1	74	1.91	0.01043	Up
marker of proliferation Ki-67	MKI67	1159	1.89	0.00712	Up
melanophilin	MLPH	122	1.92	0.00457	Down
MMS22 like, DNA repair protein	MMS22L	121	1.60	0.00111	Up
mutS homolog 5	MSH5	18	1.54	0.02348	Up
metallothionein-1A	MT1A	2897	5.65	0.00467	Down
metallothionein 1E	MT1E	1292	3.40	0.00839	Down
mitochondrial fission regulator 2	MTFR2	19	1.86	0.01106	Up
myotubularin related protein 7	MTMR7	34	1.78	0.00148	Down
myomesin 1	MYOM1	4533	1.78	0.04488	Down
non-SMC condensin I complex subunit G	NCAPG	71	1.96	0.00663	Up
NDC80 kinetochore complex component	NDC80	38	1.98	0.00471	Up
nei like DNA glycosylase 3	NEIL3	12	1.87	0.00966	Up
NIMA related kinase 2	NEK2	37	1.65	0.04376	Up
neuroligin 1	NLGN1	144	2.16	0.02588	Up
nuclear receptor subfamily 1 group D member 1	NR1D1	865	1.54	0.00668	Down
neuritin 1	NRN1	34	1.50	0.03946	Up
NUF2 component of NDC80 kinetochore complex	NUF2	55	1.94	0.00061	Up
nucleolar and spindle associated protein 1	NUSAP1	70	2.02	0.00188	Up

obscurin, cytoskeletal calmodulin and titin-interacting RhoGEF	OBSCN	83	1.61	0.02497	Down
origin recognition complex subunit 1	ORC1	27	2.38	0.00006	Up
PDZ binding kinase	PBK	40	1.72	0.03343	Up
PCNA clamp associated factor	PCLAF	51	1.91	0.01857	Up
programmed cell death 1 ligand 2	PDCD1LG2	192	1.54	0.03377	Up
prodynorphin	PDYN	86	2.53	0.01667	Up
profilin 2	PFN2	139	2.24	0.00020	Down
pepsinogen 5, group I (pepsinogen A)	PGA5	12	1.88	0.01384	Up
polo like kinase 4	PLK4	70	1.57	0.00903	Up
phospholipid transfer protein	PLTP	1020	1.79	0.04536	Up
DNA polymerase theta	POLQ	81	1.57	0.00703	Up
peroxisome proliferator activated receptor gamma	PPARG	17	1.70	0.00311	Up
periplakin	PPL	5544	1.52	0.00672	Down
protein regulator of cytokinesis 1	PRC1	83	1.79	0.01277	Up
proline rich 11	PRR11	46	1.82	0.02431	Up
pancreatic trypsin inhibitor	PTI	26	1.64	0.01934	Down
Rac GTPase activating protein 1	RACGAP1	138	1.53	0.01657	Up
RAD51 recombinase	RAD51	68	1.54	0.01333	Up
RAD51 associated protein 1	RAD51AP1	28	1.62	0.00884	Up
Ras association domain family member 6	RASSF6	25	1.60	0.00273	Up
ribonucleoprotein, PTB binding 2	RAVER2	30	1.85	0.01056	Up
RAD52 motif containing 1	RDM1	10	1.80	0.00849	Up
REC8 meiotic recombination protein	REC8	371	4.38	0.04611	Up
ribonuclease A family member 10 (inactive)	RNASE10	98	1.61	0.00041	Up
ring finger protein 150	RNF150	319	1.67	0.00339	Down
ribosomal protein L3 like	RPL3L	37	1.76	0.00046	Down
ribonucleotide reductase regulatory subunit M2	RRM2	163	1.88	0.01026	Up
ryanodine receptor 3	RYR3	21	2.06	0.02164	Down
S100 calcium binding protein A2	S100A2	1410	1.62	0.04911	Down
S100 calcium binding protein A8	S100A8	39	1.58	0.03889	Down
serum amyloid A4, constitutive	SAA4	24128	1.55	0.02860	Down
Scm polycomb group protein like 2	SCML2	23	1.50	0.01505	Up
stromal cell derived factor 2 like 1	SDF2L1	222	1.67	0.03569	Down
SEC14 like lipid binding 6	SEC14L6	429	1.59	0.01752	Down
secreted frizzled related protein 1	SFRP1	327	2.78	0.00000	Down
shugoshin 1	SGO1	33	1.90	0.00306	Up
SHC binding and spindle associated 1	SHCBP1	33	2.58	0.00024	Up
sialic acid binding Ig like lectin 15	SIGLEC15	34	1.89	0.03557	Up
spindle and kinetochore associated complex subunit 3	SKA3	43	1.51	0.01628	Up
solute carrier family 13 member 5	SLC13A5	2488	3.14	0.01353	Down
solute carrier family 22 member 16	SLC22A16	229	1.73	0.01507	Down
solute carrier family 51 subunit alpha	SLC51A	70	2.12	0.02996	Down

solute carrier family 7 member 5	SLC7A5	49	2.07	0.00234	Down
structural maintenance of chromosomes 2	SMC2	412	1.59	0.00198	Up
synuclein alpha	SNCA	119	4.11	0.00339	Down
sperm associated antigen 5	SPAG5	64	1.65	0.03242	Up
spindle apparatus coiled-coil protein 1	SPDL1	20	1.76	0.00512	Up
scaffold protein involved in DNA repair	SPIDR	935	1.64	0.04557	Down
spectrin beta, erythrocytic	SPTB	1058	1.60	0.00035	Down
stathmin 1	STMN1	170	1.87	0.00256	Up
sulfotransferase family 2B member 1	SULT2B1	26	1.87	0.01808	Down
transcription factor 19	TCF19	64	1.70	0.02213	Up
thrombospondin type 1 domain containing 7A	THSD7A	166	1.50	0.00140	Up
TOPBP1 interacting checkpoint and replication regulator	TICRR	31	1.73	0.01963	Up
toll like receptor 10	TLR10	26	1.57	0.00368	Up
tropomodulin 1	TMOD1	16	1.52	0.01469	Up
DNA topoisomerase II alpha	TOP2A	425	2.01	0.00337	Up
tumor protein p53 inducible nuclear protein 1	TP53INP1	7111	1.51	0.00112	Up
translocator protein 2	TSPO2	14	2.73	0.00035	Down
TTK protein kinase	TTK	46	1.90	0.01124	Up
titin	TTN	554	1.55	0.00092	Up
ubiquitin conjugating enzyme E2 C	UBE2C	67	1.83	0.00783	Up
unc-79 homolog, NALCN channel complex subunit	UNC79	33	1.80	0.01519	Up
wolframin ER transmembrane glycoprotein	WFS1	548	1.59	0.01210	Down
X-linked Kx blood group antigen, Kell and VPS13A binding protein	XK	87	1.55	0.04450	Up
zinc finger DHHC-type palmitoyltransferase 19	ZDHHC19	213	1.81	0.00984	Down

[1]Mid-lactation multiparous Holstein cows were retrospectively grouped as HE and LE (n = 12/group).
[2]Annotation of gene transcripts and affiliated gene symbols are based on the *Bos taurus* reference genome (release 106, ARS-UCS 1.2).
[3]Normalized mean read count as determined by DESeq2 (Love et al., 2014).
[4]Up or down expression relative to HE cow; Up = upregulated in HE compared with LE; Down = downregulated in HE compared with LE.

Supplementary Table S4. Gene Ontology domains enriched in upregulated differentially expressed genes in liver samples from cows that were high feed efficient (HE; n = 12) or low feed efficient (LE; n = 12)[1].

Gene Ontology	ID	Gene Symbols[2]
**Biological Process**		
cell division	GO:0051301	*ASPM, BIRC5, CCNA2, CCNB1, CCNB2, CCNE2, CCNF, CDC25A, CDC6, CDCA8, CDK1, ERCC6L, FBXO5, KIF11, KNL1, MASTL, NCAPG, NDC80, NUF2, SGO1, SKA3, SPAG5, SPDL1, UBE2C*
microtubule-based movement	GO:0007018	*CENPE, KIF11, KIF15, KIF18A, KIF20A, KIF22, KIF2C, KIF4A, KIFC1*
mitotic cytokinesis	GO:0000281	*ANLN, BIRC5, CEP55, CKAP2, ECT2, KIF20A, KIF4A, NUSAP1, RACGAP1*
mitotic cell cycle	GO:0000278	*ASPM, CENPE, CENPF, CENPW, KIF18A, MASTL, PLK4, SKA3*
chromosome segregation	GO:0007059	*BIRC5, CENPW, DLGAP5, HJURP, NEK2, SKA3, TTK*
G2/M transition of mitotic cell cycle	GO:0000086	*BIRC5, CCNA2, CDC25A, CDK1, FOXM1, MASTL*
mitotic metaphase plate congression	GO:0007080	*CCNB1, CDCA8, KIF18A, KIF22, KIFC1, SPDL1*
mitotic spindle assembly checkpoint	GO:0007094	*BIRC5, BUB1, BUB1B, KNTC1, SPDL1, TTK*
mitotic sister chromatid segregation	GO:0000070	*CDCA8, ESPL1, KNTC1, NEK2, SGO1*
mitotic spindle organization	GO:0007052	*CENPE, DLGAP5, KIF4A, NDC80, NUF2*
mitotic cell cycle phase transition	GO:0044772	*CCNA2, CCNB1, CCNB2, CCNE2*
mitotic DNA replication checkpoint	GO:0033314	*CDC6, CLSPN, ORC1, TICRR*
protein localization to kinetochore	GO:0034501	*CDK1, KNL1, SPDL1, TTK*
regulation of attachment of spindle microtubules to kinetochore	GO:0051988	*ECT2, NEK2, RACGAP1, SPAG5*
regulation of cyclin-dependent protein serine/threonine kinase activity	GO:0000079	*CCNA2, CCNB1, CCNB2, CCNE2*
kinetochore organization	GO:0051383	*NDC80, NUF2, SMC2*
meiotic chromosome segregation	GO:0045132	*NUF2, SGO1, SMC2*
mitotic spindle midzone assembly	GO:0051256	*KIF4A, PRC1, RACGAP1*
positive regulation of chromosome segregation	GO:0051984	*CDC6, NCAPG, SMC2*
**Cellular Component**		
nucleus	GO:0005634	*ASPM, BIRC5, CCNA2, CCNB1, CCNB2, CCNE2, CCNF, CDC25A, CDC6, CDK1, CDKN2C, CENPF, CLSPN, DEPDC1, DLGAP5, E2F7, ECT2, ESPL1, EXO1, FBXO5, FOXM1, H2B, IGF2BP3, KIF11, KIF18A, KIF20A, KIF22, KNL1, MASTL,*

		MKI67, MMS22L, NEIL3, NEK2, PBK, PCLAF, PLK4, PLTP, PPARG, PRR11, RAD51, RAD51AP1, RAVER2, SCML2, SGO1, SPDL1, TCF19, TICRR, TOP2A, TP53INP1, TTK, UBE2C
cytoplasm	GO:0005737	*ASPM, BIRC5, CCNA2, CCNB1, CCNB2, CCNE2, CCNF, CCNF, CDC25A, CDKN2C, CENPF, CEP55, CFAP43, CKAP2, CKAP2L, CLEC7A, CYP2J2, DLGAP5, DNA2, ECT2, ESPL1, FBXO5, FGF21, IGF2BP3, KIF15, KIF18A, KIF22, KNTC1, MASTL, MELK, NCAPG, NEK2, NUSAP1, PLK4, PPARG, PRC1, PRR11, RAD51, SMC2, SPDL1, STMN1, TOP2A, TTK*
nucleoplasm	GO:0005654	*ANLN, BUB1, CCNA2, CDC20, CDC6, CDCA8, CDK1, CENPE, CENPF, CENPW, CLSPN, DNA2, E2F8, ESCO2, FANCI, FBXO5, HJURP, KIF20A, KIF4A, MASTL, NDC80, NEIL3, NEK2, NUF2, ORC1, POLQ, PPARG, PRC1, RACGAP1, SGO1, SMC2, TICRR, TOP2A*
cytosol	GO:0005829	*BIRC5, BUB1, CCNA2, CDC20, CDC6, CDK1, CENPE, CIP2A, CKAP2, CKAP2L, DLGAP5, E2F8, ECT2, FANCI, GIMAP6, GIMAP8, IGF2BP3, KIF11, KIF22, KNTC1, NUF2, PLK4, POLQ, RDM1, RRM2, SGO1, SKA3, TICRR, TP53INP1, UBE2C*
centrosome	GO:0005813	*CCNB1, CCNB2, CCNE2, CDK1, CKAP2, CKAP2L, ESPL1, MASTL, NDC80, NEK2, PCLAF, PLK4, SGO1, SKA3*
kinetochore	GO:0000776	*BIRC5, BUB1, CENPE, CENPW, ERCC6L, HJURP, KIF18A, KIF2C, KNL1, NEK2, REC8, SGO1, SKA3, TTK*
midbody	GO:0030496	*ANLN, ASPM, BIRC5, CDCA8, CDK1, CENPE, CENPF, CEP55, ECT2, KIF20A, KIF4A, NEK2, PRC1, RACGAP1*
microtubule	GO:0005874	*CENPE, CKAP2, KIF15, KIF18A, KIF20A, KIF22, KIF2C, KIFC1, NUSAP1, STMN1*
spindle pole	GO:0000922	*ASPM, CDC6, CENPF, CKAP2L, KIF11, KNTC1, NEK2, PLK4, SGO1, SPDL1*
mitotic spindle	GO:0072686	*CDC6, CDK1, CKAP2L, ESPL1, KIF11, KIF22, NUSAP1, SKA3, SPAG5*
chromosome, centromeric region	GO:0000775	*BIRC5, CDCA8, CENPF, HJURP, SGO1, TOP2A*
cleavage furrow	GO:0032154	*ECT2, KIF20A, MASTL, PLK4, RACGAP1*
condensed chromosome outer kinetochore	GO:0000940	*BUB1B, CENPF, NDC80, SKA3, SPDL1*
intercellular bridge	GO:0045171	*CDC6, CDCA8, CEP55, KIF20A, PRC1*
condensed chromosome	GO:0000793	*NCAPG, SGO1, SMC2, TOP2A*
cyclin-dependent protein kinase holoenzyme complex	GO:0000307	*CCNA2, CCNB1, CCNB2, CCNE2*
nuclear chromosome	GO:0000228	*MSH5, RAD51, SMC2, TOP2A*
spindle microtubule	GO:0005876	*BIRC5, CDK1, KIF11, SKA3*
kinetochore microtubule	GO:0005828	*CENPE, KIF18A, KNTC1*
mitotic spindle midzone	GO:1990023	*CENPE, KIF18A, PRC1*

**Molecular Function**		
ATP binding	GO:0005524	*BUB1*, *BUB1B*, *CDK1*, *CENPE*, *DNA2*, *ERCC6L*, *KIF11*, *KIF15*, *KIF18A*, *KIF20A*, *KIF22*, *KIF2C*, *KIF4A*, *KIFC1*, *MASTL*, *MELK*, *MSH5*, *NEK2*, *ORC1*, *PBK*, *PLK4*, *POLQ*, *RAD51*, *SMC2*, *TOP2A*, *TTK*, *UBE2C*
microtubule binding	GO:0008017	*BIRC5*, *CENPE*, *CENPF*, *DLGAP5*, *GAS2L3*, *KIF11*, *KIF15*, *KIF18A*, *KIF20A*, *KIF22*, *KIF2C*, *KIF4A*, *KIFC1*, *NUSAP1*, *PRC1*, *RACGAP1*
chromatin binding	GO:0003682	*CDK1*, *EXO1*, *ORC1*, *PCLAF*, *POLQ*, *PPARG*, *RAD51*, *REC8*, *SCML2*, *SMC2*, *TICRR*, *TOP2A*
protein kinase binding	GO:0019901	*CCNA2*, *CDC25A*, *CDKN2C*, *FBXO5*, *FOXM1*, *KIF11*, *KIF20A*, *PRC1*, *RACGAP1*
microtubule motor activity	GO:0003777	*CENPE*, *KIF15*, *KIF18A*, *KIF20A*, *KIF22*, *KIF2C*, *KIF4A*, *KIFC1*
single-stranded DNA binding	GO:0003697	*MMS22L*, *NEIL3*, *RAD51*, *RAD51AP1*, *SMC2*
cyclin-dependent protein serine/threonine kinase regulator activity	GO:0016538	*CCNA2*, *CCNB1*, *CCNB2*, *CCNE2*
anaphase-promoting complex binding	GO:0010997	*CDC20*, *CLSPN*, *FBXO5*
kinetochore binding	GO:0043515	*CENPE*, *SPDL1*, *TTK*
pattern recognition receptor activity	GO:0038187	*CLEC4E*, *CLEC6A*, *CLEC7A*
single-stranded DNA-dependent ATP-dependent DNA helicase activity	GO:0017116	*DNA2*, *POLQ*, *RAD51*

[1]Enrichment analysis was performed using the Database for Annotation, Visualization, and Integrated Discovery (v. 2021) by comparing differentially expressed genes (mean read count ≥ 10; *P*-value ≤ 0.05; fold change ≥ 1.5). Fold enrichment and Benjamini corrected *P*-values are reported.
[2]Annotation of gene transcripts and affiliated gene symbols are based on the *Bos taurus* reference genome (release 106, ARS-UCD 1.2).

Supplemental Table S5. Differentially expressed genes in muscle tissue between high feed efficient (HE) and low feed efficient (LE) mid-lactation cows[1].

Gene[2]	Gene Symbol	Mean[3]	Fold Change	*P*-value	Expression[4]
3-hydroxybutyrate dehydrogenase 1	BDH1	37	2.68	0.00111	Down
5'-nucleotidase, cytosolic II	NT5C2	665	1.66	0.01628	Down
abhydrolase domain containing 2, acylglycerol lipase	ABHD2	1884	1.97	0.01157	Down
actin related protein 1B	ACTR1B	489	1.62	0.03951	Down
activating transcription factor 3	ATF3	2523	5.29	0.01645	Down
acyl-CoA thioesterase 11	ACOT11	28	2.75	0.00016	Down
ADAM metallopeptidase with thrombospondin type 1 motif 20	ADAMTS20	1269	1.79	0.03410	Down
ADAM metallopeptidase with thrombospondin type 1 motif 3	ADAMTS3	52	1.96	0.02738	Down
ADAM metallopeptidase with thrombospondin type 1 motif 8	ADAMTS8	17	3.38	0.03263	Up
adenosylmethionine decarboxylase 1	AMD1	952	1.54	0.04223	Down
adhesion G protein-coupled receptor L1	ADGRL1	216	1.68	0.01797	Up
ADP ribosylation factor like GTPase 4D	ARL4D	18	1.54	0.03327	Up
ADP ribosylation factor like GTPase 5B	ARL5B	793	1.51	0.00795	Down
aldolase, fructose-bisphosphate C	ALDOC	24	1.58	0.04415	Up
amphiphysin	AMPH	31	1.53	0.02850	Up
angiotensinogen	AGT	51	1.73	0.04896	Up
ankyrin repeat and BTB domain containing 2	ABTB2	166	2.16	0.02500	Down
apelin	APLN	119	1.80	0.01837	Up
apolipoprotein E	APOE	131	1.82	0.03985	Up
aquaporin 11	AQP11	12	1.60	0.04887	Up
armadillo repeat containing X-linked 3	ARMCX3	1383	1.63	0.00041	Down
arrestin domain containing 3	ARRDC3	608	1.50	0.01531	Up
arylformamidase	AFMID	15	1.58	0.04843	Up
ATPase H+ transporting V0 subunit a4	ATP6V0A4	11	2.01	0.04939	Down
ATPase Na+/K+ transporting subunit beta 1	ATP1B1	2435	1.56	0.03409	Down
BAG cochaperone 2	BAG2	1286	1.52	0.01350	Down
basic helix-loop-helix family member e23	BHLHE23	17	2.77	0.01463	Up
beta-1,4-N-acetyl-galactosaminyltransferase 1	B4GALNT1	12	2.03	0.00411	Up
bicaudal D homolog 1 (Drosophila) pseudogene	BICD1	300	2.38	0.00151	Up
BMP2 inducible kinase	BMP2K	1232	1.94	0.01545	Down
brain derived neurotrophic factor	BDNF	33	2.52	0.01751	Down
BTB domain and CNC homolog 1	BACH1	1446	1.84	0.01157	Down
calcium voltage-gated channel auxiliary subunit beta 2	CACNB2	46	1.53	0.04640	Up
calcium/calmodulin dependent protein kinase kinase 1	CAMKK1	18	1.56	0.04343	Up
calmodulin regulated spectrin associated protein family member 3	CAMSAP3	18	1.93	0.03791	Up

cAMP responsive element modulator	CREM	247	2.01	0.01434	Down
carboxypeptidase X, M14 family member 2	CPXM2	71	1.87	0.01219	Up
CASK interacting protein 1	CASKIN1	10	2.06	0.00987	Up
cathepsin K	CTSK	757	1.81	0.03354	Down
cell migration inducing hyaluronidase 2	CEMIP2	7621	1.92	0.01768	Down
ceramide synthase 4	CERS4	14	1.59	0.03086	Up
choline kinase alpha	CHKA	83	1.90	0.03461	Down
chondroadherin	CHAD	40	1.85	0.04635	Up
chromobox 8	CBX8	52	1.72	0.02139	Up
chromosome 15 C11orf52 homolog	C15H11orf52	41	2.00	0.04694	Down
coiled-coil domain containing 136	CCDC136	46	1.66	0.00946	Down
collagen triple helix repeat containing 1	CTHRC1	29	2.46	0.02718	Down
collagen type VI alpha 5 chain	COL6A5	326	10.23	0.00103	Up
complement factor B	CFB	167	3.23	0.00794	Up
connector enhancer of kinase suppressor of Ras 2	CNKSR2	525	1.58	0.02855	Down
crystallin lambda 1	CRYL1	258	1.52	0.04602	Down
CYLD lysine 63 deubiquitinase	CYLD	1385	1.56	0.03604	Down
cysteine and glycine rich protein 3	CSRP3	10548	2.38	0.04787	Down
cysteine and serine rich nuclear protein 1	CSRNP1	460	3.39	0.04830	Down
cysteine rich secretory protein LCCL domain containing 1	CRISPLD1	11	1.74	0.01258	Up
cytochrome P450 family 1 subfamily A member 1	CYP1A1	42	2.05	0.02290	Up
cytochrome P450 family 2 subfamily B member 6	CYP2B6	16	2.82	0.03958	Up
DIX domain containing 1	DIXDC1	1367	1.60	0.03347	Down
DNA damage inducible transcript 3	DDIT3	226	1.89	0.03016	Down
DNA damage inducible transcript 4	DDIT4	2751	1.90	0.01568	Up
DNA topoisomerase I	TOP1	1722	1.53	0.02389	Down
DnaJ heat shock protein family (Hsp40) member B4	DNAJB4	4198	1.89	0.03559	Down
DOT1 like histone lysine methyltransferase	DOT1L	2262	1.89	0.01708	Down
dual oxidase 2	DUOX2	22	3.02	0.01759	Up
dystrophin related protein 2	DRP2	18	2.29	0.00510	Up
EF-hand and coiled-coil domain containing 1	EFCC1	13	2.31	0.00107	Up
EF-hand domain containing 1	EFHC1	15	1.75	0.00681	Up
endoplasmic reticulum aminopeptidase 2	ERAP2	303	1.68	0.00223	Up
endothelin receptor type A	EDNRA	46	1.58	0.01576	Up
establishment of sister chromatid cohesion N-acetyltransferase 1	ESCO1	926	1.68	0.02850	Down
estrogen related receptor beta	ESRRB	105	1.88	0.00778	Down
eukaryotic translation initiation factor 4E	EIF4E	1494	1.51	0.01276	Down
exoribonuclease 1	ERI1	76	1.55	0.02274	Down
extracellular leucine rich repeat and fibronectin type III domain containing 2	ELFN2	18	2.63	0.01906	Up
FA complementation group I	FANCI	20	1.50	0.03162	Up
estrogen-induced osteoclastogenesis regulator 1	FAM102A	150	1.86	0.04810	Down

FAT atypical cadherin 3	FAT3	26	1.77	0.00927	Up
fatty acid binding protein 3	FABP3	1364	1.89	0.00983	Down
fatty acid desaturase 2	FADS2	137	1.64	0.01706	Up
FERM domain containing kindlin 1	FERMT1	28	1.52	0.03827	Up
fibrillin 3	FBN3	11	3.87	0.02412	Up
fibroblast growth factor 12	FGF12	17	1.65	0.01788	Up
fibronectin type III domain containing 1	FNDC1	166	1.62	0.02816	Up
fin bud initiation factor homolog	FIBIN	143	1.76	0.00447	Up
folliculin interacting protein 2	FNIP2	1577	3.12	0.00830	Down
forkhead box D3	FOXD3	32	1.86	0.00257	Up
frizzled class receptor 4	FZD4	872	1.50	0.02284	Up
fructose-bisphosphatase 1	FBP1	93	2.68	0.00915	Up
G protein-coupled receptor 63	GPR63	39	1.78	0.00547	Up
gasdermin B	GSDMB	56	1.92	0.02251	Up
glucosaminyl N-acetyl) transferase 4	GCNT4	67	2.10	0.01032	Down
glutamate ionotropic receptor kainate type subunit 5	GRIK5	15	1.56	0.04312	Up
glutamate ionotropic receptor NMDA type subunit 3A	GRIN3A	23	2.62	0.01383	Up
glutathione S-transferase omega 1	GSTO1	79	137.19	0.00000	Down
glycosyltransferase 1 domain containing 1	GLT1D1	194	1.98	0.01343	Down
glypican 3	GPC3	109	1.50	0.04799	Up
golgin A4	GOLGA4	16379	1.61	0.00037	Down
growth regulating estrogen receptor binding 1	GREB1	832	2.63	0.02227	Down
GTP cyclohydrolase I feedback regulator	GCHFR	23	1.81	0.00126	Up
heme oxygenase 2	HMOX2	530	2.26	0.04215	Down
hemicentin 1	HMCN1	245	2.51	0.00495	Down
hexokinase 2	HK2	9826	2.46	0.01856	Down
homeodomain interacting protein kinase 2	HIPK2	1123	1.62	0.02570	Down
hyaluronan binding protein 4	HABP4	457	1.60	0.03011	Down
immediate early response 3	IER3	589	2.25	0.02645	Down
inhibin subunit alpha	INHA	11	2.45	0.02497	Up
inositol polyphosphate multikinase	IPMK	273	2.13	0.01423	Down
insulin like growth factor 2	IGF2	4795	1.55	0.00262	Up
integrator complex subunit 6 like	INTS6L	27	1.56	0.04260	Up
interferon lambda receptor 1	IFNLR1	181	2.28	0.03777	Down
interferon related developmental regulator 1	IFRD1	1771	2.60	0.02729	Down
kelch like family member 30	KLHL30	721	2.86	0.02529	Down
kelch like family member 40	KLHL40	4554	2.70	0.02520	Down
kinectin 1	KTN1	4699	1.58	0.00037	Down
kinesin family member 1A	KIF1A	37	2.08	0.01445	Up
L1 cell adhesion molecule	L1CAM	34	1.60	0.02901	Up
leptin receptor	LEPR	108	1.53	0.02324	Up
leucine rich repeat containing 3B	LRRC3B	20	1.71	0.01691	Up

leucine rich repeat containing 4	LRRC4	11	1.96	0.00537	Up
leucine rich repeat containing 8 VRAC subunit B	LRRC8B	344	3.38	0.01111	Down
leucine rich repeat neuronal 3	LRRN3	31	2.27	0.04638	Down
LIM homeobox 6	LHX6	56	1.56	0.01153	Up
lipin 1	LPIN1	6111	1.58	0.03856	Down
LON peptidase N-terminal domain and ring finger 3	LONRF3	1515	1.73	0.02102	Down
mab-21 like 1	MAB21L1	42	1.75	0.01481	Up
macrophage stimulating 1	MST1	15	1.80	0.03731	Up
MAF bZIP transcription factor F	MAFF	1130	2.70	0.01592	Down
MAPK activated protein kinase 3	MAPKAPK3	1154	1.70	0.03634	Down
marker of proliferation Ki-67	MKI67	67	1.72	0.04741	Up
mastermind like transcriptional coactivator 3	MAML3	80	1.58	0.00187	Up
methyltransferase 21C, AARS1 lysine	METTL21C	321	3.05	0.00973	Down
minichromosome maintenance 10 replication initiation factor	MCM10	223	1.51	0.02165	Down
mitogen-activated protein kinase kinase 3	MAP2K3	1117	1.78	0.04112	Down
mohawk homeobox	MKX	11	2.07	0.03348	Up
mono-ADP ribosylhydrolase 2	MACROD2	39	1.57	0.04076	Up
monoamine oxidase B	MAOB	232	1.80	0.03808	Up
myogenin	MYOG	810	1.80	0.03481	Down
myomaker, myoblast fusion factor	MYMK	53	1.58	0.04268	Up
myosin heavy chain 8	MYH8	1900	1.59	0.02933	Up
myosin light chain kinase family member 4	MYLK4	1707	2.26	0.04413	Down
myosin VB	MYO5B	41	1.97	0.01973	Up
N-acetylglucosamine-1-phosphate transferase subunits alpha and beta	GNPTAB	2125	2.79	0.01262	Down
NCK associated protein 5	NCKAP5	16	2.31	0.03567	Up
nectin cell adhesion molecule 3	NECTIN3	401	1.60	0.03955	Down
NEDD4 binding protein 2 like 2	N4BP2L2	3972	1.52	0.03056	Down
neurotrophic receptor tyrosine kinase 2	NTRK2	343	1.51	0.01123	Up
nexilin F-actin binding protein	NEXN	15277	1.70	0.00418	Down
Nik related kinase	NRK	162	1.65	0.02241	Up
NIPA magnesium transporter 2	NIPA2	731	1.61	0.01764	Down
collagen type XXII alpha 1 chain	COL22A1	37	2.94	0.04581	Up
uncharacterized CYR61	CYR61	884	1.67	0.01003	Up
uncharacterized FAM84A	FAM84A	20	2.11	0.00649	Up
heat shock factor binding protein 1 like 1	HSBP1L1	11	1.87	0.01147	Up
uncharacterized KIAA1551	KIAA1551	971	1.57	0.02958	Down
kelch like family member 34	KLHL34	5620	1.50	0.04678	Down
uncharacterized LOC100126544	LOC100126544	29	1.56	0.03395	Down
uncharacterized LOC100196897	LOC100196897	100	2.15	0.01231	Down
uncharacterized LOC100196898	LOC100196898	14	1.67	0.04550	Down
60S ribosomal protein L31 pseudogene	LOC100297150	105	1.80	0.01455	Down

uncharacterized LOC100850659	LOC100850659	52	2.23	0.00153	Up
uncharacterized LOC101902998	LOC101902998	35	1.67	0.03715	Up
uncharacterized LOC101903290	LOC101903290	674	2.00	0.00156	Up
60S ribosomal protein L15 pseudogene	LOC101903301	34	5.45	0.00040	Down
uncharacterized LOC101903873	LOC101903873	88	1.84	0.02679	Up
transcription factor BTF3 homolog 4 pseudogene	LOC101904275	17	1.53	0.03395	Up
polycystic kidney disease and receptor for egg jelly-related protein	LOC101904413	55	1.92	0.02250	Down
zinc finger protein 160 pseudogene	LOC101905179	38	2.20	0.00000	Down
tetraspanin-3 pseudogene	LOC101906398	19	1.91	0.00824	Up
uncharacterized LOC101907117	LOC101907117	117	1.81	0.02960	Up
tropomyosin alpha-3 chain-like	LOC104970173	44	1.55	0.02558	Up
uncharacterized LOC104970622	LOC104970622	13	2.14	0.00195	Up
uncharacterized LOC104970902	LOC104970902	17	2.01	0.03768	Down
uncharacterized LOC104971707	LOC104971707	18	3.37	0.00584	Down
polycystic kidney disease and receptor for egg jelly-related protein-like	LOC104972622	12	2.14	0.02448	Down
uncharacterized LOC104975960	LOC104975960	12	1.77	0.02849	Down
uncharacterized LOC104976281	LOC104976281	3396	1.90	0.02302	Down
sialic acid-binding Ig-like lectin 14	LOC107131224	12	2.26	0.00625	Down
uncharacterized LOC107132478	LOC107132478	13	3.38	0.00291	Up
uncharacterized LOC107132490	LOC107132490	25	1.59	0.01235	Down
uncharacterized LOC107132649	LOC107132649	13	1.97	0.01210	Down
uncharacterized LOC107132707	LOC107132707	46	1.77	0.03500	Down
uncharacterized LOC107133222	LOC107133222	12	1.65	0.04303	Down
uncharacterized LOC112441829	LOC112441829	26	1.53	0.03662	Up
uncharacterized LOC112441863	LOC112441863	35	4.02	0.00211	Down
uncharacterized LOC112442189	LOC112442189	37	1.77	0.01053	Up
small nucleolar RNA SNORA16B/SNORA16A family	LOC112443648	37	1.54	0.03077	Up
U4atac minor spliceosomal RNA	LOC112443671	590	1.67	0.03663	Up
40S ribosomal protein S24 pseudogene	LOC112444773	24	1.92	0.01497	Down
uncharacterized LOC112446006	LOC112446006	71	1.87	0.02397	Down
small Cajal body-specific RNA 4	LOC112446113	30	1.62	0.03197	Up
small nucleolar RNA SNORA23	LOC112446862	170	1.60	0.00780	Up
pregnancy zone protein	LOC506828	34	2.95	0.01775	Up
keratin-associated protein 5-1	LOC515676	28	7.90	0.00004	Up
tyrosine-protein phosphatase non-receptor type 11	LOC526769	96	2.98	0.02912	Down
cystatin-13	LOC531692	76	1.97	0.00648	Down
vascular cell adhesion molecule 1-like	LOC534578	24	2.17	0.00950	Up
dnaJ homolog subfamily A member 3, mitochondrial pseudogene	LOC534627	55	2.05	0.04292	Down
glyceraldehyde-3-phosphate dehydrogenase pseudogene	LOC615002	64	1.63	0.00774	Up
glyceraldehyde-3-phosphate dehydrogenase pseudogene	LOC617654	89	1.73	0.00133	Up

retinoic acid early transcript 1E	LOC785982	249	4.15	0.03084	Down
uncharacterized LOC787122	LOC787122	108	2.17	0.01563	Up
40S ribosomal protein S23	LOC787803	106	1.86	0.00745	Up
aflatoxin B1 aldehyde reductase member 4	LOC788425	37	1.50	0.02347	Up
uncharacterized LOC790886	LOC790886	35	4.42	0.00113	Up
purinergic receptor P2Y13	P2RY13	27	1.74	0.02830	Down
selenophosphate synthetase 2	SEPHS2	925	2.02	0.04997	Down
tigger transposable element derived 4	TIGD4	31	2.44	0.01049	Up
NPC intracellular cholesterol transporter 1	NPC1	699	2.52	0.02481	Down
nuclear receptor subfamily 4 group A member 3	NR4A3	5015	33.59	0.00000	Down
nucleotide binding oligomerization domain containing 2	NOD2	43	2.36	0.00213	Down
nudE neurodevelopment protein 1 like 1	NDEL1	1362	1.62	0.03397	Down
oxidative stress responsive serine rich 1	OSER1	1441	1.54	0.04686	Down
peroxisome proliferator activated receptor delta	PPARD	1035	2.73	0.01145	Down
phosphatidylinositol specific phospholipase C X domain containing 2	PLCXD2	153	1.64	0.03047	Down
phosphatidylinositol-3,4,5-trisphosphate dependent Rac exchange factor 1	PREX1	390	1.62	0.02513	Down
phosphodiesterase 4D	PDE4D	5506	1.89	0.01800	Down
phosphoenolpyruvate carboxykinase 2, mitochondrial	PCK2	21	2.46	0.02093	Up
phospholipase C beta 1	PLCB1	251	1.99	0.00097	Down
Pim-1 proto-oncogene, serine/threonine kinase	PIM1	837	2.06	0.04619	Down
podoplanin	PDPN	143	2.07	0.04026	Down
potassium voltage-gated channel subfamily Q member 1	KCNQ1	10	1.84	0.00766	Up
PPARG coactivator 1 alpha	PPARGC1A	3623	4.99	0.00495	Down
prolyl 4-hydroxylase subunit alpha 3	P4HA3	67	2.22	0.01487	Down
protein kinase AMP-activated non-catalytic subunit gamma 2	PRKAG2	1103	2.75	0.00911	Down
protein kinase cGMP-dependent 2	PRKG2	13	2.25	0.02153	Up
protein phosphatase 1 regulatory subunit 15A	PPP1R15A	2035	4.13	0.02116	Down
protein phosphatase 1 regulatory subunit 36	PPP1R36	16	1.66	0.01261	Down
protein phosphatase targeting COQ7	PPTC7	1816	1.64	0.04237	Down
protein tyrosine phosphatase non-receptor type 3	PTPN3	359	2.03	0.00956	Down
proteoglycan 4	PRG4	68	2.88	0.03739	Down
RAB15 effector protein	REP15	76	2.08	0.02689	Down
RAR related orphan receptor C	RORC	100	1.57	0.02111	Up
Ras association domain family member 4	RASSF4	44	1.52	0.03843	Up
Ras association domain family member 8	RASSF8	1191	1.78	0.00361	Down
regulator of calcineurin 2	RCAN2	238	1.65	0.02370	Down
regulator of G protein signaling 6	RGS6	133	1.51	0.01216	Up
regulatory factor X2	RFX2	154	2.63	0.02881	Down
reticulophagy regulator 1	RETREG1	5351	3.17	0.00658	Down
retinol binding protein 4	RBP4	222	1.90	0.02902	Up

retinyl ester hydrolase type 1	BREH1	18	1.84	0.04565	Up
Rho associated coiled-coil containing protein kinase 2	ROCK2	8062	1.84	0.00926	Down
ribonuclease A family member 10 (inactive)	RNASE10	68	1.50	0.02444	Up
ribosome biogenesis regulator 1 homolog	RRS1	239	1.66	0.01718	Down
ring finger protein 115	RNF115	1208	1.75	0.02029	Down
ring finger protein 41	RNF41	381	1.75	0.03614	Down
RNA binding motif protein 20	RBM20	5137	1.66	0.03788	Down
RNA binding motif protein 7	RBM7	339	1.50	0.02124	Down
ROS proto-oncogene 1, receptor tyrosine kinase	ROS1	17	2.48	0.04220	Down
RRAD, Ras related glycolysis inhibitor and calcium channel regulator	RRAD	2085	2.31	0.03694	Down
RUNX family transcription factor 1	RUNX1	487	2.63	0.03183	Down
S100 calcium binding protein A9	S100A9	34	2.17	0.03981	Down
secreted phosphoprotein 2	SPP2	15	2.75	0.00140	Up
serine protease 53	PRSS53	35	1.50	0.00183	Up
SH3 domain containing ring finger 3	SH3RF3	61	1.62	0.02537	Up
shroom family member 2	SHROOM2	21	2.20	0.00908	Up
shroom family member 3	SHROOM3	122	2.13	0.00943	Up
slit guidance ligand 2	SLIT2	50	1.66	0.03342	Down
small nucleolar RNA SNORA63	LOC112448305	13	2.12	0.00051	Up
small nucleolar RNA SNORA70	LOC112443079	34	1.84	0.00457	Up
small nucleolar RNA SNORA70	LOC112447183	25	1.77	0.00999	Up
small nucleolar RNA SNORA71	LOC112449443	92	1.83	0.00162	Up
small nucleolar RNA SNORD116	LOC112443258	17	1.55	0.02234	Up
small nucleolar RNA SNORD116	LOC112443276	19	2.00	0.00849	Up
small nucleolar RNA SNORD14	LOC112441683	17	2.95	0.00021	Up
small nucleolar RNA SNORD36	LOC112448904	39	1.88	0.00166	Up
small nucleolar RNA U3	LOC112442867	787	1.77	0.01535	Up
small nucleolar RNA Z195/SNORD33/SNORD32 family	LOC112442484	75	1.58	0.03124	Up
small nucleolar RNA SNORD89	LOC112448929	1149	1.57	0.01018	Up
small nucleolar RNA SNORA12	LOC112444563	39	1.59	0.00809	Up
small nucleolar RNA SNORA31	LOC112449172	52	1.76	0.00646	Up
small nucleolar RNA SNORA49	LOC112442094	52	1.52	0.00586	Up
small nucleolar RNA SNORA52	LOC112444967	45	2.41	0.00019	Up
solute carrier family 16 member 5	SLC16A5	556	1.64	0.02743	Down
solute carrier family 25 member 33	SLC25A33	106	2.30	0.01357	Down
solute carrier family 28 member 1	SLC28A1	11	3.16	0.03886	Down
solute carrier family 38 member 4	SLC38A4	88	3.23	0.01223	Up
sperm antigen with calponin homology and coiled-coil domains 1	SPECC1	2936	1.59	0.02420	Down
sphingomyelin phosphodiesterase 3	SMPD3	67	3.37	0.00232	Up
stearoyl-CoA desaturase	SCD	7005	1.64	0.01950	Up
sterile alpha motif domain containing 14	SAMD14	21	1.80	0.00382	Up

sterile alpha motif domain containing 15	SAMD15	60	1.76	0.01030	Down
sushi, nidogen and EGF like domains 1	SNED1	129	1.77	0.02597	Down
syndecan 4	SDC4	1646	3.70	0.01159	Down
teneurin transmembrane protein 4	TENM4	124	1.83	0.02028	Up
testis specific serine kinase 3	TSSK3	16	7.04	0.03740	Down
thioredoxin interacting protein	TXNIP	24995	1.62	0.00327	Up
thrombospondin 4	THBS4	641	2.30	0.00082	Up
thrombospondin type 1 domain containing 4	THSD4	34	1.61	0.03917	Up
thyroid hormone responsive	THRSP	55	2.45	0.03276	Up
TNF receptor associated factor 1	TRAF1	321	2.61	0.00062	Up
transcobalamin 1	TCN1	13	2.74	0.02890	Down
transcriptional and immune response regulator	TCIM	175	1.57	0.04423	Up
transmembrane protein 120B	TMEM120B	880	1.54	0.03678	Down
transmembrane protein 251	TMEM251	176	1.80	0.00746	Down
tripartite motif containing 54	TRIM54	7030	1.53	0.02863	Down
tumor protein p53 inducible nuclear protein 2	TP53INP2	1336	1.69	0.02068	Down
U1 spliceosomal RNA	LOC112449629	36	2.44	0.00425	Up
U2 spliceosomal RNA	LOC112442853	90	2.83	0.00621	Up
U4 spliceosomal RNA	LOC112442110	578	1.88	0.00249	Up
U4 spliceosomal RNA	LOC112442111	464	1.68	0.01278	Up
U6 spliceosomal RNA	LOC112448206	26	1.88	0.02750	Down
ubiquitin specific peptidase 50	USP50	49	1.84	0.01140	Down
VANGL planar cell polarity protein 2	VANGL2	15	1.55	0.03462	Up
vascular endothelial growth factor A	VEGFA	2618	1.96	0.02116	Down
vasohibin 1	VASH1	114	1.51	0.01678	Up
von Willebrand factor A domain containing 2	VWA2	24	2.70	0.00485	Up
Wnt ligand secretion mediator	WLS	959	1.76	0.02305	Down
xin actin binding repeat containing 1	XIRP1	62013	4.79	0.00395	Down
X-prolyl aminopeptidase 2	XPNPEP2	23	2.12	0.00165	Up
zinc finger and BTB domain containing 10	ZBTB10	1019	1.57	0.02276	Down
zinc finger and BTB domain containing 43	ZBTB43	848	2.06	0.01602	Down
zinc finger protein 385B	ZNF385B	162	2.42	0.00150	Down
zinc finger protein 774	ZNF774	554	1.90	0.01718	Down
zinc finger RANBP2-type containing 1	ZRANB1	2647	1.51	0.01023	Down
zinc finger SWIM-type containing 4	ZSWIM4	243	2.42	0.03391	Down

[1]Mid-lactation multiparous Holstein cows were retrospectively grouped as HE and LE (n = 8/group).
[2]Annotation of gene transcripts and affiliated gene symbols are based on the *Bos taurus* reference genome (release 106, ARS-UCS 1.2).
[3]Normalized mean read count as determined by DESeq2 (Love et al., 2014).
4Up or down expression relative to HE cows; Up = upregulated in HE compared with LE; Down = downregulated in HE compared with LE.

Supplementary Table S6. Gene Ontology domains enriched in differentially expressed genes in muscle samples from cows that were high feed efficient (HE; n = 8) or low feed efficient (LE; n = 8)[1].

Gene Ontology	ID	Gene Symbols[2]
Upregulated in HE		
Cell Component: extracellular space	GO:0005615	*AGT, APLN, APOE, CHAD, COL6A5, CPXM2, CRISPLD1, ELFN2, FZD4, IGF2, INHA, LOC506828, LOC515676, LRRC3B, MST1, RBP4, THBS4, VASH1, VWA2*
Molecular Function: actin filament binding	GO:0051015	*CACNB2, CAMSAP3, FERMT1, MYH8, MYO5B, SAMD14, SHROOM2, SHROOM3*
Downregulated in HE		
Cell Component: nucleus	GO:0005634	*ARMCX3, ATF3, BACH1, BMP2K, CREM, CSRNP1, CSRP3, DDIT3, DOT1L, EIF4E, ESCO1, ESRRB, FABP3, HABP4, HIPK2, IER3, IFRD1, IPMK, LPIN1, MAFF, MAPKAPK3, MCM10, METTL21C, MYOG, N4BP2L2, NR4A3, PDE4D, PLCB1, PPARD, PPARGC1A, PRKAG2, RBM20, RBM7, RCAN2, RFX2, ROCK2, RUNX1, S100A9, TP53INP2, ZBTB10, ZBTB43, ZNF385B, ZNF774, ZRANB1*

[1]Enrichment analysis was performed using the Database for Annotation, Visualization, and Integrated Discovery (v. 2021) by comparing differentially expressed genes (mean read count ≥ 10; *P*-value ≤ 0.05; fold change ≥ 1.5). Fold enrichment and Benjamini corrected *P*-values are reported.

[2]Annotation of gene transcripts and affiliated gene symbols are based on the *Bos taurus* reference genome (release 106, ARS-UCD 1.2).
